# Observational methods for COVID-19 vaccine effectiveness research: an empirical evaluation and target trial emulation

**DOI:** 10.1093/ije/dyad138

**Published:** 2023-10-13

**Authors:** Martí Català, Edward Burn, Trishna Rathod-Mistry, Junqing Xie, Antonella Delmestri, Daniel Prieto-Alhambra, Annika M Jödicke

**Affiliations:** Centre for Statistics in Medicine, Nuffield Department of Orthopaedics, Rheumatology and Musculoskeletal Sciences, University of Oxford, Oxford, UK; Centre for Statistics in Medicine, Nuffield Department of Orthopaedics, Rheumatology and Musculoskeletal Sciences, University of Oxford, Oxford, UK; Centre for Statistics in Medicine, Nuffield Department of Orthopaedics, Rheumatology and Musculoskeletal Sciences, University of Oxford, Oxford, UK; Centre for Statistics in Medicine, Nuffield Department of Orthopaedics, Rheumatology and Musculoskeletal Sciences, University of Oxford, Oxford, UK; Centre for Statistics in Medicine, Nuffield Department of Orthopaedics, Rheumatology and Musculoskeletal Sciences, University of Oxford, Oxford, UK; Centre for Statistics in Medicine, Nuffield Department of Orthopaedics, Rheumatology and Musculoskeletal Sciences, University of Oxford, Oxford, UK; Department of Medical Informatics, Erasmus University Medical Center, Rotterdam, The Netherlands; Centre for Statistics in Medicine, Nuffield Department of Orthopaedics, Rheumatology and Musculoskeletal Sciences, University of Oxford, Oxford, UK

**Keywords:** COVID-19 vaccines, COVID-19, methods research, epidemiology, propensity scores, vaccine effectiveness, electronic heath records, overlap weighting, inverse probability of treatment weighting, matching

## Abstract

**Background:**

There are scarce data on best practices to control for confounding in observational studies assessing vaccine effectiveness to prevent COVID-19. We compared the performance of three well-established methods [overlap weighting, inverse probability treatment weighting and propensity score (PS) matching] to minimize confounding when comparing vaccinated and unvaccinated people. Subsequently, we conducted a target trial emulation to study the ability of these methods to replicate COVID-19 vaccine trials.

**Methods:**

We included all individuals aged ≥75 from primary care records from the UK [Clinical Practice Research Datalink (CPRD) AURUM], who were not infected with or vaccinated against SARS-CoV-2 as of 4 January 2021. Vaccination status was then defined based on first COVID-19 vaccine dose exposure between 4 January 2021 and 28 January 2021. Lasso regression was used to calculate PS. Location, age, prior observation time, regional vaccination rates, testing effort and COVID-19 incidence rates at index date were forced into the PS. Following PS weighting and matching, the three methods were compared for remaining covariate imbalance and residual confounding. Last, a target trial emulation comparing COVID-19 at 3 and 12 weeks after first vaccine dose vs unvaccinated was conducted.

**Results:**

Vaccinated and unvaccinated cohorts comprised 583 813 and 332 315 individuals for weighting, respectively, and 459 000 individuals in the matched cohorts. Overlap weighting performed best in terms of minimizing confounding and systematic error. Overlap weighting successfully replicated estimates from clinical trials for vaccine effectiveness for ChAdOx1 (57%) and BNT162b2 (75%) at 12 weeks.

**Conclusion:**

Overlap weighting performed best in our setting. Our results based on overlap weighting replicate previous pivotal trials for the two first COVID-19 vaccines approved in Europe.

Key Messages

- Real-world evidence was generated using weighting (overlapping weights and inverse probability of treatment weights) and propensity score matching, with our study successfully replicating the findings of phase 3 trials for COVID-19 vaccine effectiveness.
- Overlap weighting provides the least biased estimates in our study and should be considered among the most suitable methods for future COVID-19 vaccine effectiveness research.
- Despite a lack of trial data, our findings suggest that first-dose BNT162b2 provides effective protection against SARS-COV-2 infection for up to 12 weeks, in line with UK’s Joint Committee on Vaccination and Immunisation modelling and subsequent vaccination strategies.

## Introduction

Routinely collected data are widely used to evaluate the effectiveness and safety of COVID-19 vaccines^1–4^ and boosters.^5^^,^^6^ However, careful consideration of how to best account for confounding is required when comparing vaccinated and unvaccinated people: patient characteristics such as age and comorbidities associated with high risk of severe COVID-19, as well as population-level information such community transmission and local vaccination rates at a time, are among the most important confounders. However, the latter were not always adequately considered in previously conducted vaccine effectiveness studies. Differences in methods for confounding adjustment, choice of study design, inclusion criteria and calendar time can have substantial impact on study findings. Recent observational studies^7^^,^^8^ assessing the comparative effectiveness of ChAdOx1 (Oxford/AstraZeneca) and BNT162b2 (BioNTech/Pfizer) reported a 28% decreased risk for infection after vaccination with BNT162b2(8) vs no differences in infection incidence.^7^ Another challenge when studying vaccine effectiveness is the handling of the immediate time after the first vaccine dose. Randomized trials^9^^,^^10^ showed no protective vaccine effect in the first 2 weeks, with the vaccine-induced immunity still building up. However, whereas some observational studies replicated these findings,^1^ others already observed protective effects in the early days,^11^^,^^12^ indicating the presence of residual confounding. Some studies and even trials omitted this vulnerable time from their main analyses^10^ and others did not,^9^ which makes it difficult to compare results across studies. Several methods were used in previous studies, but a rigorous assessment of their ability to resolve confounding has not yet been completed.^13^ Moreover, studies benchmarking vaccine effectiveness findings from observational studies to the ‘gold standard’ of phase 3 randomized controlled trials (RCT) are scarce.

### Objectives and manuscript structure

Our study comprises two overarching objectives. Objective 1 aimed to empirically evaluate the comparative performance of three different methods to minimize confounding in the study of COVID-19 vaccine effectiveness: overlap weighting (OW),^14^ inverse probability of treatment weights (IPTW)^15^ and propensity score (PS) with exact geographical and index date matching.

We (i) calculated PS, which represent the probability of exposure based on a participant’s baseline characteristics. We used different representations of geographical location, as we deemed this to be a key confounder for our subsequent analyses. After applying weighting and matching techniques, we (ii) compared our methods with respect to (a) remaining confounding based on imbalances between baseline characteristics in vaccinated vs unvaccinated subjects, (b) statistical power based on minimum detectable relative risk and (c) systematic error due to unobserved confounders using control outcomes.

Objective 2 aimed to conduct target trial emulations for the phase III randomized controlled trials which assessed effectiveness for the two different vaccine brands that were available first in England:^16^ BNT162b2 and ChAdOx1.

We (i) used the preferred method from objective 1, (ii) calculated risk for COVID-19 at 3 and 12 weeks after first vaccine dose vs unvaccinated, and subsequently (iii) compared our findings with results from RCTs. As no trials assessed the effect of BNT162b2 between 3 and 12 weeks following the first dose, we estimated vaccine effectiveness based on primary care data and compared our results with the models the Joint Committee on Vaccination and Immunisation (JCVI) used to inform the vaccination campaign in the UK in early 2021.^17^

## Methods

A glossary of terms and methods is provided in the Supplementary Material (available as Supplementary data at *IJE* online).

###  

#### Study type, setting and data source

We conducted a cohort study using UK primary care data from the Clinical Practice Research Datalink (CPRD) AURUM, mapped to the Observational Medical Outcomes Partnership (OMOP) Common Data Model (CDM).^18^

#### Study population

We included people with high risk for severe COVID-19, i.e. aged ≥75 years, who were prioritized for vaccination according to the UK Government’s Priority Groups for vaccination. Among those, people with no previous record of COVID-19 [diagnosis/positive polymerase chain reaction (PCR) test, no previous COVID-19 vaccination, and with data availability for ≥180days before study start (4 January 2021)] were included to the source population.

#### Exposure: COVID-19 vaccination

**Figure 1.**
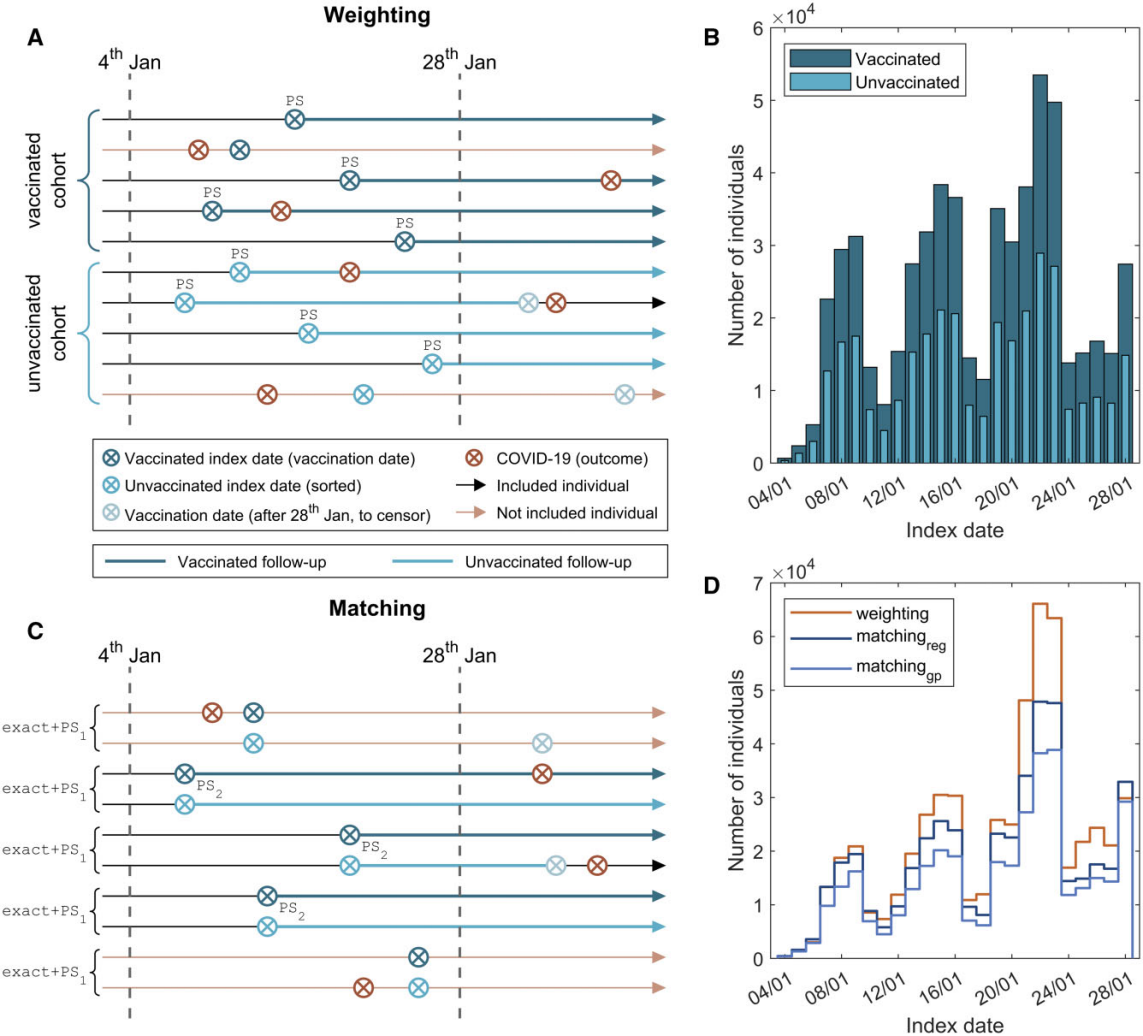
Index date and follow-up in vaccinated and unvaccinated cohorts. (A) Diagram depicting index date and follow-up for both vaccinated and unvaccinated cohorts in weighted analyses; exclusion based on COVID-19 infection status. Follow-up is represented by a thicker/wider solid arrow. (B) Distribution of index dates for vaccinated (dark blue) and unvaccinated cohorts (light blue). (C) Diagram depicting index date and follow-up for both vaccinated and unvaccinated participants in matched analyses; exclusion based on COVID-19 infection status. Follow-up period is represented by a thicker line. (D) Distribution of index dates for weighted and different matched analyses

From the source population, individuals were classified as vaccinated (VC) or unvaccinated (UV), based on whether they received a first COVID-19 vaccine dose in the period between 4 January 2021 and 28 January 2021, when this age group was eligible and prioritized for vaccination in the UK. We constructed three vaccinated cohorts, namely any vaccination, ChAdOx1 vaccinated, and BNT162b2 vaccinated cohorts. Index date for vaccinated people was their vaccination date. Individuals with a record of both vaccine brands at index date were excluded. Index date for unvaccinated people was either the index date of the matched vaccinated counterpart [for matching] or was randomly assigned following the distribution of index dates in the vaccinated cohort [for weighting] to account for immortal time bias^19^ (Figure 1 and Supplementary Figure S2, available as Supplementary data at *IJE* online). After assignment of index dates, individuals with a recording of COVID-19 infection before or on index date were excluded.

#### COVID-19 definition

Two definitions were used for COVID-19: COVID-19 PCR was a positive COVID-19 PCR test, and COVID-19 PCR/diagnosis was a COVID-19 clinical diagnosis or a positive COVID-19 test (Supplementary Tables S2–S4, available as Supplementary data at *IJE* online).

### Minimizing confounding in vaccine effectiveness research

#### Propensity scores

Covariates to be included in the large-scale PS were selected from conditions and drugs recorded in three time windows (1–30 days, 31–180 days and 181 days to any time prior index date), and two time windows (1–30 days, 31–180 days prior index date), respectively. Among those, covariates with a frequency >0.5% in the study population were included in a lasso regression, which was then used for variable selection.^20^^,^^21^ A clinical review of the selected covariates was conducted to exclude instrumental variables. In addition, pre-defined key confounders identified from the literature were directly forced into the PS model: location [region identifier or General Practice (GP) surgery identifier]; age [continuous and categorical (5-year bands) to account for non-linearity]; prior observation years; number of outpatient visits; and number of previous COVID-19 PCR tests.

#### PS weighting and matching

For weighting, regional vaccination, testing and COVID-19 incidence rates^22^ on index date, plus index date itself, were forced into the PS. PS were computed using logistic regression, with three different representations of location included: without location (PS_base_), region (PS_reg_) or de-identified GP surgery (PS_gp_). We used two weighting methods: inverse probability of treatment weighting (IPTW), with trimming over 0.95 and below 0.05 to reduce the impact of extreme weights; and overlap weighting (OW), which down-weights the tails of the PS distribution and emphasizes the population with highest overlap in observed characteristics between treatments.^23^

For matching, we used 1:1 PS nearest neighbours matching, combining exact matching for age band, gender and location (region or GP) with caliper matching (width 0.2 standard deviation) for PS (PS_1_). After unvaccinated people were assigned the index date of their matched vaccinated counterpart, PS were computed again on the new index date (PS_2_) to ensure that the matching was still balanced after index date assignment.

#### Comparison of model performance to minimize confounding

The following metrics were used to assess the performance of the different methods.

##### Baseline covariate imbalance

Covariate imbalance between vaccinated vs unvaccinated cohorts after weighting/matching was used to assess ‘measured confounding’, with absolute standardized mean differences (SMD) >0.1 indicating imbalance.^24^

##### Statistical power

Minimum detectable relative risk (MDRR) was computed for a 95% confidence interval, power of 0.8, a 10-day cumulative incidence of 0.0067 and 10 days of follow-up time to indicate statistical power for the respective methods.

##### Control outcomes

- Although negative control outcomes (NCO) are outcomes not causally associated with the exposure of interest,^25^ here COVID-19 vaccination, their association with vaccination should be affected by the same type of unmeasured confounding, e.g. health care-seeking behaviour, as the vaccination-outcome association.^26^ In all, 43 NCOs were pre-specified based on previous methodological research on vaccine safety^27^ and refined after review by two clinical epidemiologists [D.P.A., A.M.J.] (Supplementary Table S4, available as Supplementary data at *IJE* online). Significant associations between NCO with vaccination in our study would indicate unmeasured confounding.
- Diagnostic effort: we used PCR testing, i.e. performed COVID-19 PCR test regardless of test result, in the first 10 days after index date as a control outcome and proxy for diagnostic effort. Differences in diagnostic effort associated with vaccination would indicate residual bias.
- Replication of null effect of vaccine effectiveness in first 10 days: association between vaccination status and COVID-19 PCR, COVID-19 PCR/diagnosis in the first 10 days after index date was estimated to detect unmeasured confounding through comparison with results from RCT.^9^

Associations between vaccination status and control outcomes were estimated using Cox proportional hazard regression. Proportionality of hazards was tested using visual inspection of Kaplan–Meier plots (Supplementary Figures S10 and S11, available as Supplementary data at *IJE* online). Hazard ratios calculated for (ii) and (iii) were empirically calibrated based on results from NCO analyses (Schuemie *et al*.^28^).

### Target trial emulation

Target trial specification is used to prevent immortal time bias when designing observation studies.^29^ We used the target trial emulation framework to reproduce the effectiveness observed at 3 and 12 weeks after vaccination in the BNT162b2 and ChAdOx1 phase 3 RCTs respectively^9^^,^^10^ and the estimated effectiveness at 12 weeks for BNT162b2. Unlike observational studies, RCTs do not distinguish between average treatment effect (ATE), average treatment in the treated (ATT) or average treatment effect in the overlap (ATO). We used the method which performed best in terms of minimizing confounding in Objective 1 for the trial emulation, irrespective of whether those methods generated ATE (i.e. IPTW), ATT (i.e. matching) or ATO (i.e. OW) estimates. A table comparing the target trial with our study is included in the Supplementary Table S5 (available as Supplementary data at *IJE* online). We prioritized synchronizing calendar time for our trial emulation, as previous studies found high variability in vaccine response according to predominant variants.^30^^,^^31^ Per-protocol analyses were conducted. People were followed up from their index date until the earliest of: receiving another vaccine dose (vaccinated cohort); or receiving a first vaccine dose (unvaccinated cohort); or departure from the database, death or 3 weeks/12 weeks following index date. Supplementary Figure S1 (available as Supplementary data at *IJE* online) illustrates this for RCTs and our study. For the ChAdOx1 trial emulation, individuals who tested positive or were censored before the 21st day were eliminated from the analysis in line with the trial’s statistical analysis plan and protocol.^10^ Cox proportional hazard regression was used to calculate hazard ratios for COVID-19 PCR and COVID-19 PCR/diagnosis, and estimates were calibrated based on NCO. We deemed a trial successfully replicated when the confidence intervals from RCT overlapped with the point estimate from observational data, or vice versa (‘coverage’).

As no trials assessed the effect of BNT162b2 between 3 and 12 weeks following the first dose, we compared our results with the models the JCVI used to inform the vaccination campaign in the UK in early 2021.^17^

## Results

### Study population and characteristics

**Figure 2.**
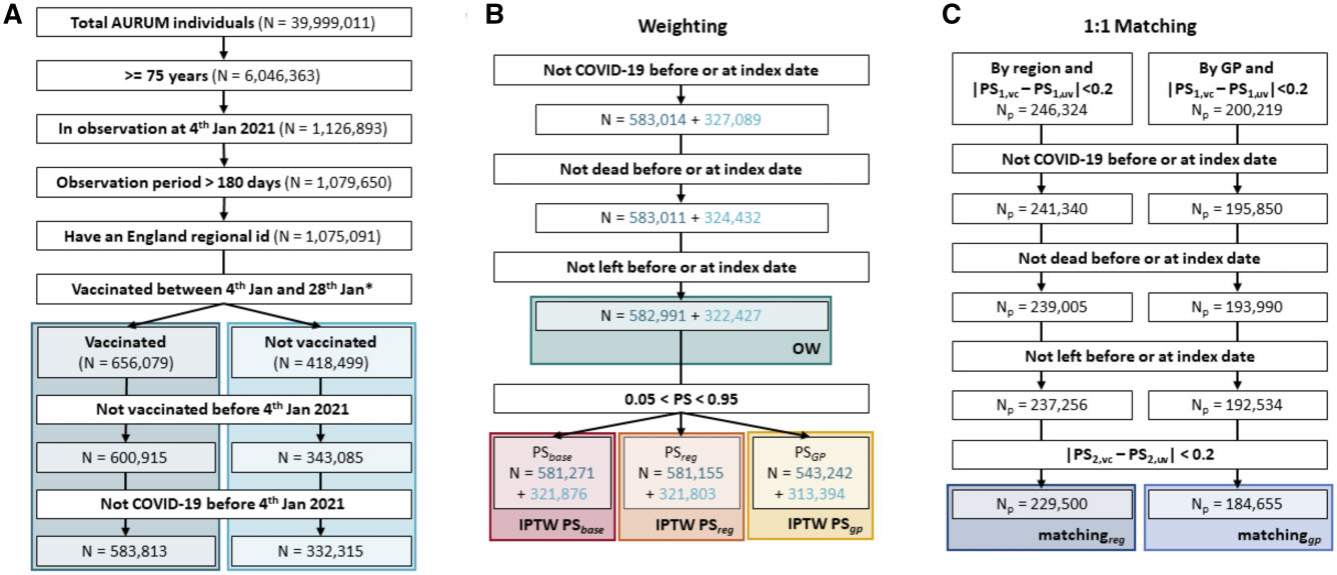
Cohorts building flowchart in any type of vaccination and unvaccinated comparison. (A) Flowchart to build the any type of vaccination (VC) and unvaccinated (UV) initial cohorts. (B) Flowchart to build the different weighting cohorts: the starting point of these cohorts is the end of panel A. Dark blue numbers are for vaccinated cohort and light for unvaccinated. PS_base:_ propensity scores were computed without location, PS_reg:_ PS were computed with region included as covariate; PS_gp:_ PS included GP practice as covariate. (C) Flowchart to build the different matching cohorts; the starting point of these cohorts is the end of panel A. PS_1_ and PS_2_ are the propensity scores (PS) computed at the start and index date, respectively. *At this step, individuals with a record of both ChAdOx1 and BNT162b2 vaccines at the index date were excluded. CPRD AURUM, Clinical Practice Research Datalink AURUM; IPTW, inverse probability treatment weighting; PS: propensity score; ChAdOx1 and BNT162b2, COVID-19 vaccines

We included 583 813 vaccinated (348 275 BNT162b2 and 235 538 ChAdOx1) and 332 315 unvaccinated people (Figure 2). OW retained the largest sample size, with a total of 905 418 participants. Trimming and the exclusion of unmatched people reduced sample size for IPTW (IPTW-PS_base_*N* = 903 147) and matching (*N*_reg_ = 459 000, *N*_gp_ = 369 310). Population flowcharts stratified by vaccine brand are available in Supplementary Figures S2 and S3 (available as Supplementary data at *IJE* online).

Baseline characteristics before weighting/matching are shown in Table 1. Relevant imbalances (SMD >0.1) between vaccinated and unvaccinated cohorts included age, location, number of outpatients visits and some comorbidities such as heart disease, hypertensive disorder, malignant neoplastic disease and renal impairment. Supplementary Tables S6 and S8 (available as Supplementary data at *IJE* online) provide details on imbalanced covariates (*N* between 23 and 27), stratified for vaccine brand.

**Table 1.** Baseline differences between unvaccinated and vaccinated cohorts before weighting/matching

Characteristic	Unvaccinated (*n*=332 315)	Vaccinated					
		Any type (*n* = 583 813)	**SMD** ^d^	**ChAdOx1** ^e^ **(*n* = 235 538)**	**SMD** ^d^	**BNT162b2** ^e^ **(*n* = 348 275)**	**SMD** ^d^
Age (years), median [IQR]	78 [76–83]	81 [78–86]	0.285	80 [77–85]	0.208	82 [78–86]	0.341
Age group			0.509		0.310		0.655
75 to 79 years	207 710 (62.5%)	224 388 (38.4%)		111 630 (47.4%)		112 758 (32.4%)	
80 to 84 years	58 693 (17.7%)	183 171 (31.4%)		61 821 (26.2%)		121 350 (34.8%)	
85 to 89 years	36 015 (10.8%)	110 762 (19.0%)		34 455 (14.6%)		76 307 (21.9%)	
90 to 94 years	21 070 (6.3%)	49 449 (8.5%)		19 299 (8.2%)		30 150 (8.7%)	
95 to 99 years	7332 (2.2%)	13 887 (2.4%)		7005 (3.0%)		6882 (2.0%)	
>99 years	1495 (0.4%)	2156 (0.4%)		1328 (0.6%)		828 (0.2%)	
Male sex	147 234 (44.3%)	252 761 (43.3%)	0.020	99 268 (42.1%)	0.044	153 493 (44.1%)	0.005
Region^a^			0.141		0.204		0.148
North East	10 595 (3.2%)	18 834 (3.2%)		8026 (3.4%)		10 808 (3.1%)	
North West	54 543 (16.4%)	119 038 (20.4%)		47 890 (20.3%)		71 148 (20.4%)	
Yorkshire and the Humber	10 580 (3.2%)	21 895 (3.8%)		11 221 (4.8%)		10 674 (3.1%)	
East Midlands	8079 (2.4%)	10 205 (1.7%)		4350 (1.8%)		5855 (1.7%)	
West Midlands	61 028 (18.4%)	106 134 (18.2%)		41 550 (17.6%)		64 584 (18.5%)	
East of England	12 591 (3.8%)	28 832 (4.9%)		12 785 (5.4%)		16 047 (4.6%)	
South West	48 825 (14.7%)	79 330 (13.6%)		36 369 (15.4%)		42 961 (12.3%)	
South East-Central	80 764 (24.3%)	125 969 (21.6%)		51 225 (21.7%)		74 744 (21.5%)	
London	45 310 (13.6%)	73 576 (12.6%)		22 122 (9.4%)		51 454 (14.8%)	
GP surgery^a^			0.725		0.815		0.974
Different GP surgeries	1355	1356		1352		1343	
Individuals per GP surgery, median [IQR]	169 [87–319]	324 [151–593]		114 [46–227]		173 [71–348]	
Prior observation time (years), median [IQR]	23.1 [9.6–34.9]	25.3 [11.4–37.1]	0.065	24.2 [9.8–36.3]	0.033	25.9 [12.6–37.7]	0.086
GP visits, median [IQR]^b^	9 [4–17]	22 [12–36]	0.624	22 [12–36]	0.634	22 [12–34]	0.617
Number of PCR tests, mean ± SD^b^	0.1±0.6	0.3±1.7	0.141	0.6±2.2	0.211	0.2±1.2	0.072
Comorbidities^c^							
Asthma	38 193 (11.5%)	70 385 (12.1%)	0.017	28 329 (12.0%)	0.017	42 056 (12.1%)	0.018
Autoimmune disease	13 920 (4.2%)	26 281 (4.5%)	0.015	10 893 (4.6%)	0.021	15 388 (4.4%)	0.011
COPD	28 627 (8.6%)	52 176 (8.9%)	0.011	21 619 (9.2%)	0.020	30 557 (8.8%)	0.006
Dementia	18 340 (5.5%)	37 944 (6.5%)	0.041	20 147 (8.6%)	0.119	17 797 (5.1%)	0.018
Diabetes mellitus	64 137 (19.3%)	112 685 (19.3%)	0.000	45 403 (19.3%)	0.001	67 282 (19.3%)	0.000
Heart disease	101 742 (30.6%)	211 270 (36.2%)	0.118	83 292 (35.4%)	0.101	127 978 (36.7%)	0.130
Hypertensive disorder	185 562 (55.8%)	355 249 (60.8%)	0.102	141 100 (59.9%)	0.082	214 149 (61.5%)	0.115
Malignant neoplastic disease	76 186 (22.9%)	160 017 (27.4%)	0.103	61 967 (26.3%)	0.079	98 050 (28.2%)	0.120
Renal impairment	80 367 (24.2%)	169 014 (29.0%)	0.108	66 110 (28.1%)	0.088	102 904 (29.5%)	0.121

The different covariates included in this table are computed at the start date (4 January 2021) for each individual.

COPD, chronic obstructive pulmonary disease; IQR, interquartile range; PCR, polymerase chain reaction; SD, standard deviation.

a Region and general practice (GP) surgery are the location identifiers.

b GP visits and number of PCR tests are only recorded for the past 180 days.

c Comorbidities are recorded for any time prior.

d Standardized mean differences (SMDs) are computed compared with the unvaccinated cohort.

e ChAdOx1 and BNT162b2 are COVID-19 vaccines.

### Minimizing confounding in vaccine effectiveness research

#### Comparison of model performance to minimize confounding

**Figure 3.**
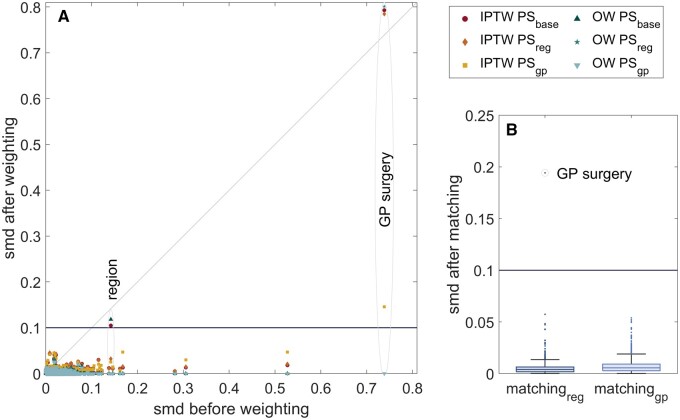
Standardized mean differences (SMD) for the different methods in any type of vaccination and unvaccinated comparison. (A) Scatter plot to compare covariate SMDs before and after PS weighting. Region and GP surgery are the only covariates that often remained unbalanced after weighting. (B) Boxplot for covariate SMDs after matching. Only GP surgery remained unbalanced after regional matching. IPTW: Inverse probability treatment weighting, OW: Overlap weighting, PS: Propensity Score, GP: General practitioner, Representations of location included in PS: ‘base’: without location, ‘region’: Region, ‘GP’: de-identified GP surgery

*Baseline covariate imbalance*: covariate imbalance after PS matching/weighting showed lower maximum SMDs in OW and matching compared with IPTW (Table 2). Including GP practice further reduced confounding, with maximum SMDs decreasing from 0.79 in IPTW-PS_base_ to 0.15 in IPTW-PS_gp_. Consequently, OW-PS_gp_ and matching_gp_ performed best, and covariate balance for GP practice was only achieved for these two models (Figure 3). Supplementary Figures S5 and S6 (available as Supplementary data at *IJE* online) show covariate balance for BNT162b2 and ChAdOx1 vs unvaccinated comparisons.

**Table 2.** Minimum detectable relative risk, standardized mean differences and significant negative control outcomes

**Method** ^a^	**Minimum detectable relative risk** ^b^	**Standardized mean difference** ^c^		**Significant (*P*<0.05) negative control outcomes** ^d^	
		Mean±SD	Maximum	*n* (%) with RR>1	*n* (%) with RR<1
Any type of vaccinated comparison					
Unweighted	<0.93; >1.08	0.023 ± 0.029	0.74	32 (74%)	1 (2%)
IPTW PS_base_	<0.93; >1.08	0.004 ± 0.019	0.79	21 (49%)	7 (16%)
IPTW PS_reg_	<0.93; >1.08	0.004 ± 0.018	0.78	20 (47%)	6 (14%)
IPTW PS_gp_	<0.92; >1.08	0.004 ± 0.005	0.15	26 (60%)	6 (14%)
OW PS_base_	<0.93; >1.08	0.002 ± 0.019	0.81	8 (19%)	4 (9%)
OW PS_reg_	<0.93; >1.08	0.002 ± 0.019	0.80	8 (19%)	4 (9%)
OW PS_gp_	<0.93; >1.08	0.002 ± 0.002	0.03	8 (19%)	3 (7%)
Matching_reg_	<0.87; >1.15	0.005 ± 0.006	0.19	17 (40%)	4 (9%)
Matching_gp_	<0.86; >1.17	0.007 ± 0.006	0.05	17 (40%)	3 (7%)
ChAdOx1 vaccinated comparison					
Unweighted	<0.86; >1.16	0.023 ± 0.029	0.82	32 (74%)	1 (2%)
IPTW PS_base_	<0.86; >1.16	0.003 ± 0.020	0.85	16 (37%)	4 (9%)
IPTW PS_reg_	<0.86; >1.16	0.003 ± 0.020	0.84	17 (40%)	4 (9%)
IPTW PS_gp_	<0.86; >1.16	0.003 ± 0.004	0.13	17 (40%)	4 (9%)
OW PS_base_	<0.86; >1.16	0.002 ± 0.020	0.86	5 (12%)	1 (2%)
OW PS_reg_	<0.86; >1.16	0.002 ± 0.020	0.85	5 (12%)	1 (2%)
OW PS_gp_	<0.86; >1.16	0.001 ± 0.002	0.02	5 (12%)	2 (5%)
Matching_reg_	<0.85; >1.18	0.004 ± 0.006	0.13	10 (23%)	2 (5%)
Matching_gp_	<0.82; >1.21	0.005 ± 0.005	0.04	10 (23%)	2 (5%)
BNT162b2 vaccinated comparison					
Unweighted	<0.89; >1.12	0.024 ± 0.036	0.99	30 (70%)	1 (2%)
IPTW PS_base_	<0.90; >1.12	0.003 ± 0.024	1.04	19 (44%)	4 (9%)
IPTW PS_reg_	<0.90; >1.12	0.003 ± 0.024	1.03	18 (42%)	4 (9%)
IPTW PS_gp_	<0.89; >1.12	0.003 ± 0.005	0.18	26 (60%)	5 (12%)
OW PS_base_	<0.89; >1.12	0.002 ± 0.025	1.05	9 (21%)	2 (5%)
OW PS_reg_	<0.89; >1.12	0.002 ± 0.024	1.04	8 (19%)	2 (5%)
OW PS_gp_	<0.89; >1.12	0.001 ± 0.002	0.03	8 (19%)	3 (7%)
Matching_reg_	<0.85; >1.18	0.005 ± 0.007	0.22	19 (44%)	2 (5%)
Matching_gp_	<0.83; >1.21	0.008 ± 0.008	0.07	17 (40%)	3 (7%)

a ‘base’, ‘region’ and ‘GP’ are different representations of location.

b Minimum detectable relative risk (range).

c Standardized mean differences (mean and SD, and maximum for all the identified covariates).

d Number of significant negative control outcomes (positive and negative; regarding if they are positively or negatively associated with the exposure).

IPTW, inverse probability treatment weighting; OW, overlap weighting; PS, propensity scores; RR, relative risk; SD, standard deviation.

*Statistical power*: minimum detectable relative risks (MDRR) are reported in Table 2. MDRR indicate higher statistical power (estimates closer to one) for weighting compared with matching, which reflects the larger sample sizes for weighting and smaller study population for matching due to the exclusion of unmatched people (Figure 2).

**Figure 4.**
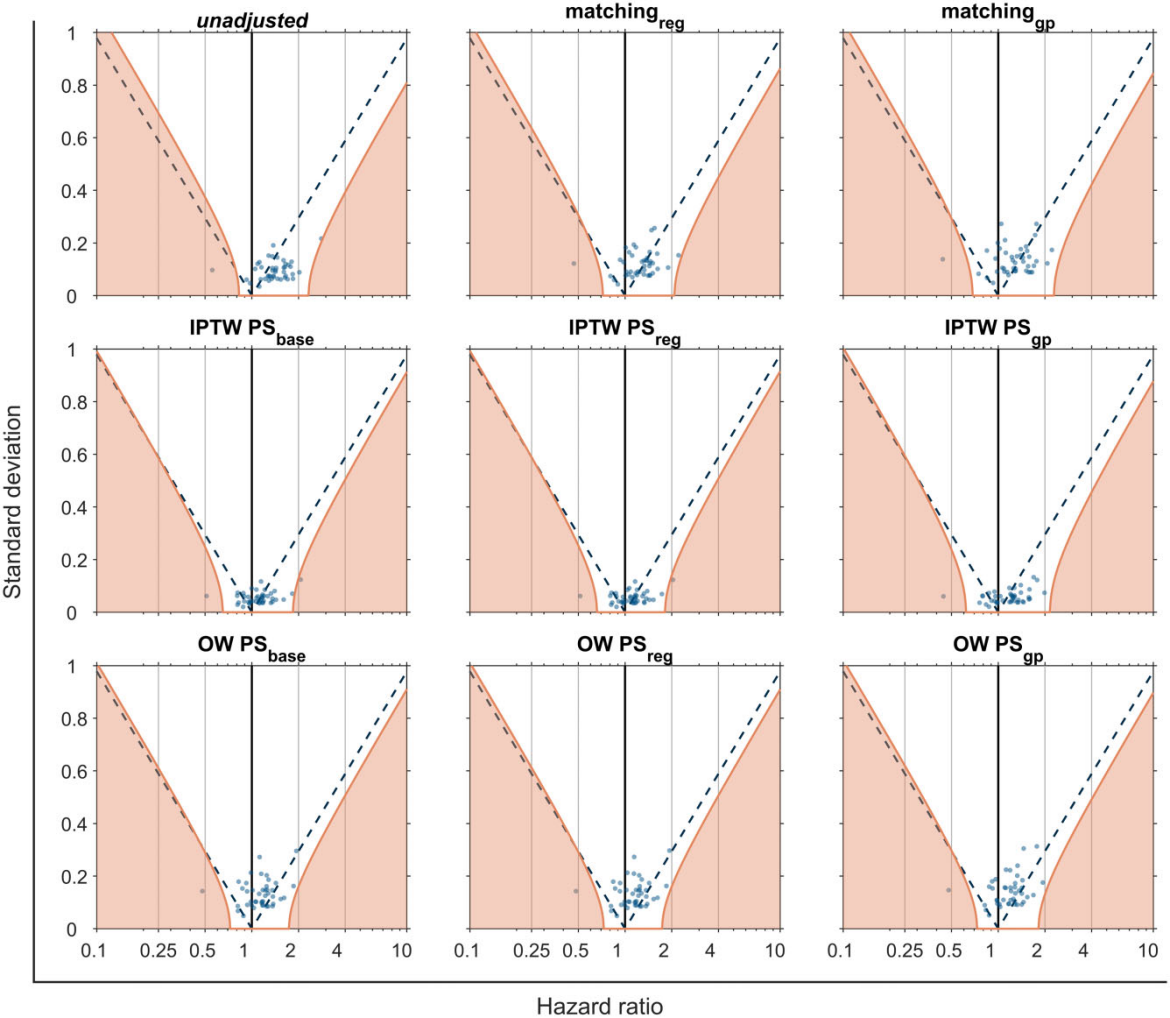
Negative control outcomes (NCO) hazard ratios and standard deviation. Each blue dot represents a different NCO. Purple dashed lines indicate the significative threshold for the NCO; they are positively correlated if they are on the right of the dashed line and negatively on the left. Orange lines mark significance thresholds after calibration, where we adjust the significant thresholds according to the negative control outcome distribution. IPTW: Inverse probability treatment weighting, OW: Overlap weighting, PS: Propensity Score, GP: General practitioner, Representations of location included in PS: ‘base’: without location, ‘region’: Region, ‘GP’: de-identified GP surgery

*Control outcomes*: as expected, clear evidence of one-sided systematic error was seen before weighting/matching, with many NCOs being positively associated (RR >1) with vaccine exposure (Table 2). NCO analyses after weighting/matching showed lower systematic error after OW compared with IPTW and matching in most scenarios (Figure 4). Supplementary Figures S7 and S8 (available as Supplementary data at *IJE* online) contain results from NCO analyses for BNT162b2 and ChAdOx1 vs unvaccinated comparisons.

**Figure 5.**
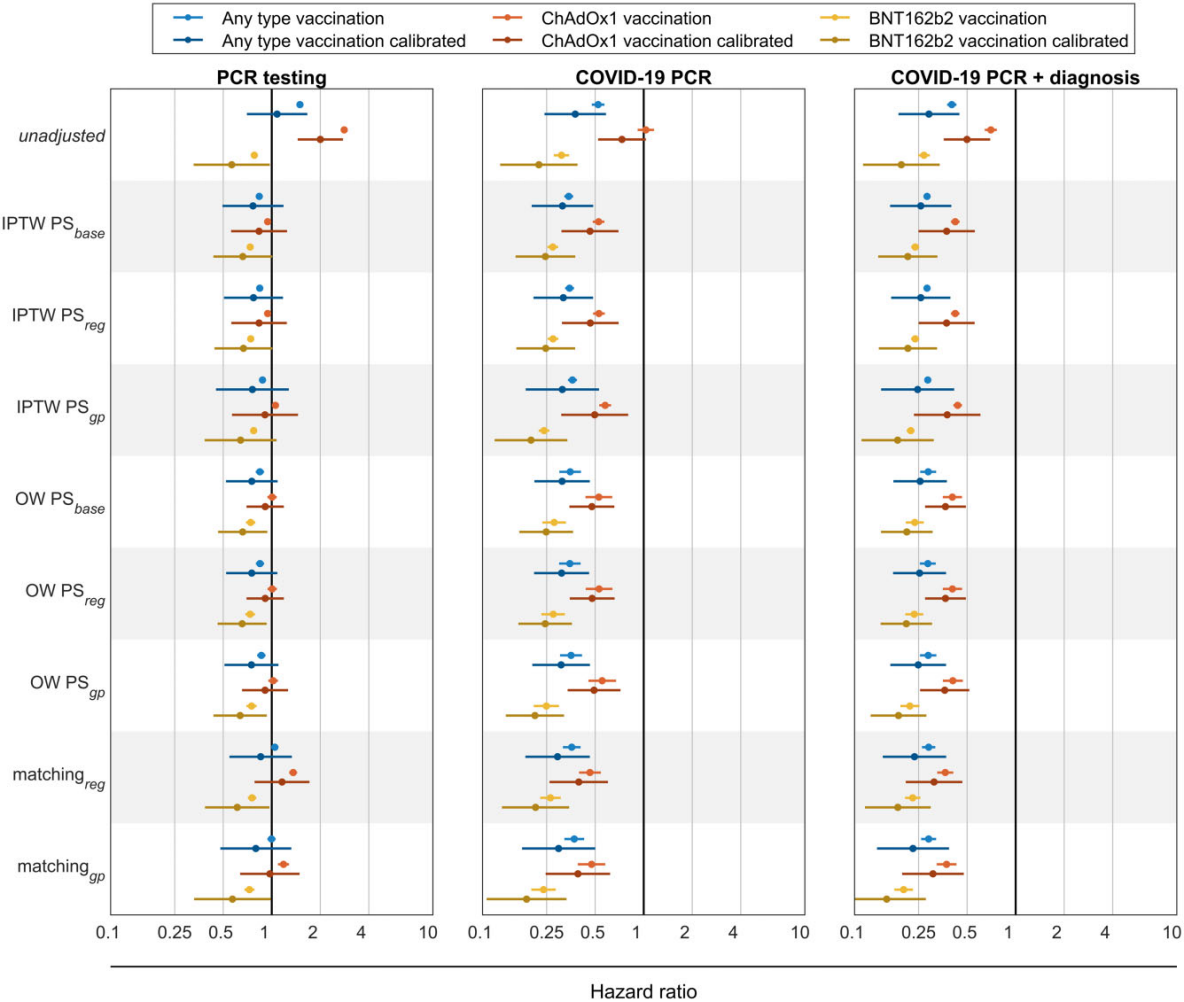
Hazard ratio for the control outcomes censoring at day 10. Each dot is the hazard ratio (HR) for a different adjustment computed with a Cox proportional hazards regression. Blue lines are for any type of vaccination compared with unvaccinated, red lines for ChAdOx1 vaccinated compared with unvaccinated and yellow lines are for BNT162b2 vaccinated compared with unvaccinated. Darker lines are for calibrated hazard ratios. Vertical black line marks the HR = 1 threshold. In the left panel, HR are for PCR testing, in the central and right panel for different COVID-19 definitions: only PCR-positive and PCR-positive or a diagnosis, respectively. IPTW, inverse probability treatment weighting; OW, overlap weighting; PS, propensity score; PCR, polymerase chain reaction; GP, general practitioner. Representations of location included in PS: ‘base’: without location, ‘region’: Region, ‘GP’: de-identified GP surgery, ChAdOx1 and BNT162b2: COVID-19 vaccines

In addition to pre-specified NCO analyses, Figure 5 shows the observed HR for the control outcome of PCR testing. Although unweighted analyses led to a biased estimate (HR >1) for PCR testing, all weighting and matching methods resolved this for any vaccination and ChAdOx1 with calibrated HR including the expected null effect (HR = 1). However, whereas BNT162b2-vaccinated people were less tested in the first 10 days compared with unvaccinated counterparts, this difference vanished when comparing testing during all available follow-up (Supplementary Figure S9, available as Supplementary data at *IJE* online).

Unexpectedly, all tested methods showed a protective effect against the clinical outcomes of PCR+ and PCR+/diagnosis in the first 10 days after first-dose vaccination (Figure 5, Supplementary Table S9, available as Supplementary data at *IJE* online). Sensitivity analyses without censoring at 10 days show similar patterns (Supplementary Figure S9 and Table S10, available as Supplementary data at *IJE* online). Kaplan–Meier plots using OW-PS_gp_ and stratified for vaccine brand are provided in Supplementary Figures S10 and S11 (available as Supplementary data at *IJE* online).

### Target trial emulation

**Figure 6.**
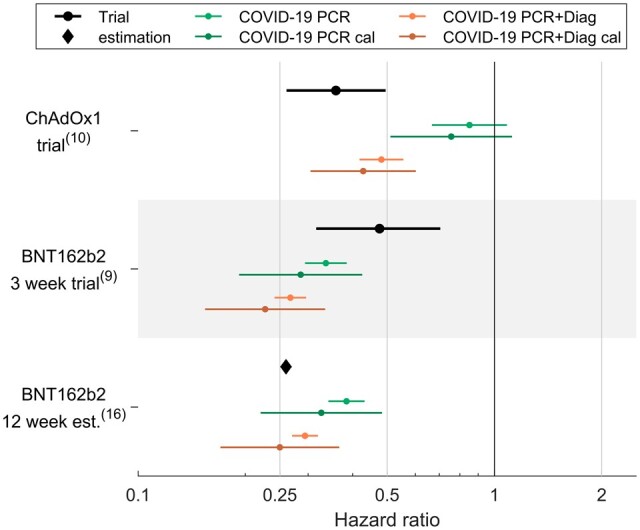
Trial emulation. Calibrated and uncalibrated relative risk for COVID-19 PCR and COVID-19 PCR and/or diagnosis. For ChAdOx1 trial, individuals with a positive test within the first 21 days were eliminated. BNT162b2 at 12 weeks estimates were provided by the Joint Committee on Vaccination and Immunisation (JCVI); however, no confidence intervals were reported. PCR, polymerase chain reaction; Diag, COVID-19 diagnosis; cal, calibrated; ChAdOx1 and BNT162b2, COVID-19 vaccines

Estimates from observational data in Figure 6 and in Supplementary Table S11 (available as Supplementary data at *IJE* online) were obtained using OW_gp_, the method yielding lowest confounding and better statistical power. For ChAdOx1, results for vaccine effectiveness were successfully replicated for COVID-19 PCR/diagnosis as outcome definition, but not for COVID-19 PCR alone. For BNT162b2, calibrated observational estimates replicated the 12-week results, with point estimates again being closer to JCVI-reported estimates for COVID-19 PCR/diagnosis than COVID-19 PCR only. HRs indicated higher effectiveness for BNT162b2 at Week 3 in our study compared with RCT findings.

## Discussion

In this large cohort study using UK primary care records, we assessed the effectiveness of a first dose of the COVID-19 vaccines BNT162b2 and ChAdOx1 using overlap weighting. Our estimates of vaccine effectiveness are in line with trial estimates for the ChAdOx1 vaccine (57%), as well as the JCVI estimates for BNT162b2 at 12 weeks (75%).

We chose OW for the target trial emulation, following an extensive investigation of the performance of different methods to account for confounding when studying COVID-19 vaccine effectiveness. Notably, merely including individual-level patient characteristics in the PS did not guarantee balance of population-level geographical variables, such as geographical region and GP, regardless of subsequent PS approaches (matching/weighting). Importantly for COVID-19 vaccine effectiveness, OW was the best performing method to minimize confounding at the population level, including geographical location, which has important implications due to known differences in the spread of SARS-CoV-2 during the study period. Additionally, NCOs demonstrated a better resolution of residual confounding with OW compared with other matching and weighting methods. Therefore, the proposed theoretical advantages of OW,^14^^,^^23^ including exact balance of measured patient characteristics and good retainment of sample size, were supported by this empirical study.

Previous early vaccine effectiveness studies using observational data overlooked the adjustment of geographical information.^32^^,^^33^ Our study, consistent with previous methodological literature, showed that geographical distributions between the vaccinated and unvaccinated individuals were markedly different,^34^ emphasizing the need for adjustment. In addition, the insufficient covariate balance after applying conventional methods highlighted the importance of the proposed diagnostics in observational studies of vaccine effectiveness. More importantly, our findings did not support the previous suspicion that less active health-seeking behaviour in unvaccinated people might confound the vaccine effectiveness observed in real-world data,^12^ as no association was found between vaccination and PCR testing in our study after empirical calibration. Besides, more unvaccinated people were tested in the first 10 days compared with people vaccinated with BNT162b2. The observed protective vaccine effects shortly after vaccination were in line with previous findings from observational data,^11^^,^^12^ which might be attributable to differences in personal behaviour during the period immediately before and after vaccination, e.g. mask wearing, avoiding crowded indoor places/shielding, rescheduling vaccination if feeling unwell, which unfortunately was unlikely to be captured by electronic health records. These protective effects might partly explain why our trial emulation did not show coverage for BNT162b2 at 3 weeks, but found higher vaccine effectiveness compared with RCT. Differences in target population, community transmission over time, and geographical location might have contributed to this.

Previous studies found little evidence for relevant heterogeneity of vaccine effectiveness with respect to age, but a high variability in vaccine response for variants.^30^^,^^31^ Moreover, community transmission, social distancing rules and herd immunity affected risk for SARS-CoV-2 infection. We therefore opted to conduct our trial emulation study among those people who were prioritized for vaccination in the community (people aged ≥75), allowing for the shortest difference in calendar time to when pivotal RCTs were conducted. This allowed us to emulate as much as possible the conditions when these RCTs were conducted, including predominant variant, herd immunity status in the population and the presence and/or intensity of non-pharmacological interventions.

We acknowledge the following limitations. Whereas testing for COVID-19 was widely available during our study period, it was reserved for hospitalized patients in the early days of the pandemic. Therefore, people might have had COVID-19 but were not diagnosed or tested. Although we excluded the first COVID-19 wave from our study period and used two different definitions to define COVID-19 as our outcome, some degree of misclassification cannot be ruled out. In addition, our study population deviated from the population included in clinical trials, particularly with respect to age. Differences in outcome definition, i.e. symptomatic COVID-19 assessed in the trials vs positive test s/clinical diagnoses in our study, are being acknowledged.

## Conclusion

Our study found a first vaccine dose against COVID-19 associated with a 69% reduced risk of COVID-19 for both the ChAdOx1 and BNT162b2 vaccines. These data confirm the estimation by JCVI that one dose of BNT162b2 provides protection beyond the 3-week period studied in pivotal trials. Second, we demonstrate that PS-based overlap weighting can replicate pivotal trials and therefore provide reliable estimates of vaccine effectiveness. Further, overlap weights performed better than IPTW or matching in controlling for measured and unmeasured covariates while retaining sample size in our setting, and could be proposed as the preferred method for future vaccine effectiveness studies. Finally, our findings illustrate the need to incorporate patient location (e.g. GP practice or region of residence) and related variables (e.g. testing and transmission rates) to minimize community- as well as patient-level confounding in the study of COVID-19 vaccine effectiveness.

## Ethics approval

The study protocol was approved through the CPRD’s Research Data Governance Process (data access CPRD) Protocol No 21_000557. The protocol can be made available upon the journal’s request.

## Supplementary Material

dyad138_Supplementary_Data

## Data Availability

Data were obtained from CPRD under the CPRD multistudy licence held by the University of Oxford after Research Data Governance (RDG) approval. Direct data sharing is not allowed, but data can be accessed from CPRD subject to protocol approval. All analysis code is available from the corresponding author upon request.
