# Supplementary material for: Observational methods for COVID-19 vaccine effectiveness research: an empirical evaluation and target trial emulation
Source: Int J Epidemiol. 2023 Oct 13;53(1):dyad138. doi: 10.1093/ije/dyad138 (PMC10859138; doi:10.1093/ije/dyad138)
Supplement: dyad138_Supplementary_Data [file dyad138_supplementary_data.docx]

**Supplementary material:**

**Observational methods for COVID-19 vaccine effectiveness research: a trial emulation and empirical evaluation**

**Martí Català, Edward Burn, Trishna Rathod-Mistry, Junqing Xie, Antonella Delmestri,**

**Daniel Prieto-Alhambra, Annika M. Jödicke**

1. **Glossary of terms/methods**

| **Term** | **Detailed description of term/method** | **Reference** |
| --- | --- | --- |
| Propensity score (PS) | Propensity Scores describe the probability of a person to be assigned to a particular treatment based on a set of observed covariates, which is typically assessed at the time of treatment assignment (i.e. at baseline). | Rosenbaum PR, Rubin DB. The central role of the propensity score in observational studies for causal effects. Biometrika 1983;79:516–24. |
| Inverse probability of treatment weighting (IPTW) | In IPTW, people are being assigned a weight based on the inverse probability of receiving treatment. With a population being assigned weights, a synthetic sample is created in which baseline covariates are balanced between treatment groups. | Austin PC, Stuart EA. Moving towards best practice when using inverse probability of treatment weighting (IPTW) using the propensity score to estimate causal treatment effects in observational studies. Stat Med. 2015;34(28):3661-79. |
| Overlap weighting (OW) | For overlap weighting, each person is assigned a weight that is proportional to the probability of that person to belonging to the opposite treatment group.  With this, treated people are weighted by the probability of not receiving treatment (1 − PS) and untreated people are weighted by the probability of receiving the treatment (PS).  The advantage of OW is that the assigned weights are smaller for extreme PS values compared to weights assigned using conventional weighting methods (such as IPTW). With this, outliers who are nearly always treated (PS near 1) or never treated (PS near 0) do not dominate results, but people whose characteristics are compatible with both receiving or not receiving treatment – the “overlap group” - contribute more. | Thomas LE, Li F, Pencina MJ. Overlap Weighting: A Propensity Score Method That Mimics Attributes of a Randomized Clinical Trial. JAMA. 2020;323(23):2417-8. |
| Empirical calibration | Based on estimates from a sample of negative controls, an empirical null distribution can be derived and used to calibrate *P* values and confidence intervals, accounting for both random and systematic error. | Schuemie MJ, Hripcsak G, Ryan PB, Madigan D, Suchard MA. Empirical confidence interval calibration for population-level effect estimation studies in observational healthcare data. Proc Natl Acad Sci U S A. 2018;115(11):2571-7. |
| Target trial emulation | Target trial emulation framework is used to design observational trials, aiming to emulating a hypothetical pragmatic randomized trial. This approach can help to prevent biases, e.g. immortal time bias, that may be introduced when failing to align start of follow-up, eligibility, and treatment assignment. | Hernan MA, Sauer BC, Hernandez-Diaz S, Platt R, Shrier I. Specifying a target trial prevents immortal time bias and other self-inflicted injuries in observational analyses. J Clin Epidemiol. 2016;79:70-5. |

1. **Clinical definition and concept list**

**Table S1.** Concept id for Polymerase chain reaction (PCR)

| **Name** | **id** | **Class** | **Domain** | **Vocabulary** |
| --- | --- | --- | --- | --- |
| Coronavirus nucleic acid detection | 44789510 | Procedure | Measurement | SNOMED |

**Table S2.** Concept ids for Polymerase chain reaction (PCR) results included in PCR+ clinical definition.

| **Name** | **id** | **Class** | **Domain** | **Vocabulary** |
| --- | --- | --- | --- | --- |
| Positive | 9191 | Qualifier value | Meas value | SNOMED |
| Detected | 4126681 | Qualifier value | Meas value | SNOMED |
| Present | 4181412 | Qualifier value | Meas value | SNOMED |
| Present | 45879438 | Answer | Meas value | LOINC |
| Positive | 45884084 | Answer | Meas value | LOINC |
| Detected | 45877985 | Answer | Meas value | LOINC |

**Table S3.** List of concept id included in COVID-19 clinical diagnosis definition.

| **Name** | **Id** | **Class** | **Domain** | **Vocabulary** |
| --- | --- | --- | --- | --- |
| Suspected coronavirus infection | 45763724 | Context-dependent | Observation | SNOMED |
| Pneumonia due to Severe acute respiratory syndrome coronavirus | 40479642 | Clinical Finding | Condition | SNOMED |
| Severe acute respiratory syndrome of upper respiratory tract | 37396171 | Clinical Finding | Condition | SNOMED |
| COVID-19 | 37311061 | Clinical Finding | Condition | SNOMED |
| Suspected COVID-19 | 37311060 | Context-dependent | Observation | SNOMED |
| Myocarditis due to disease caused by Severe acute respiratory syndrome coronavirus 2 | 37310287 | Clinical Finding | Condition | SNOMED |
| Infection of upper respiratory tract caused by Severe acute respiratory syndrome coronavirus 2 | 37310286 | Clinical Finding | Condition | SNOMED |
| Encephalopathy due to disease caused by Severe acute respiratory syndrome coronavirus 2 | 37310284 | Clinical Finding | Condition | SNOMED |
| Gastroenteritis caused by SARS-CoV-2 (severe acute respiratory syndrome coronavirus 2) | 37310283 | Clinical Finding | Condition | SNOMED |
| Otitis media due to disease caused by Severe acute respiratory syndrome coronavirus 2 | 37310254 | Clinical Finding | Condition | SNOMED |
| Pneumonia caused by Human coronavirus | 37016927 | Clinical Finding | Condition | SNOMED |
| Disease due to Coronaviridae | 4100065 | Clinical Finding | Condition | SNOMED |
| Lower respiratory infection caused by SARS-CoV-2 | 3663281 | Clinical Finding | Condition | SNOMED |
| Asymptomatic SARS-CoV-2 | 3662381 | Clinical Finding | Condition | SNOMED |
| Fever caused by Severe acute respiratory syndrome coronavirus 2 | 3661885 | Clinical Finding | Condition | SNOMED |
| Acute kidney injury due to disease caused by Severe acute respiratory syndrome coronavirus 2 | 3661748 | Clinical Finding | Condition | SNOMED |
| Thrombocytopenia due to Severe acute respiratory syndrome coronavirus 2 | 3661632 | Clinical Finding | Condition | SNOMED |
| Lymphocytopenia due to Severe acute respiratory syndrome coronavirus 2 | 3661631 | Clinical Finding | Condition | SNOMED |
| Pneumonia caused by SARS-CoV-2 | 3661408 | Clinical Finding | Condition | SNOMED |
| Acute respiratory distress syndrome due to disease caused by Severe acute respiratory syndrome coronavirus 2 | 3661406 | Clinical Finding | Condition | SNOMED |
| Acute bronchitis caused by SARS-CoV-2 | 3661405 | Clinical Finding | Condition | SNOMED |
| Dyspnea caused by Severe acute respiratory syndrome coronavirus 2 | 3656669 | Clinical Finding | Condition | SNOMED |
| Conjunctivitis due to disease caused by Severe acute respiratory syndrome coronavirus 2 | 3656668 | Clinical Finding | Condition | SNOMED |
| Cardiomyopathy due to disease caused by Severe acute respiratory syndrome coronavirus 2 | 3656667 | Clinical Finding | Condition | SNOMED |
| Rhabdomyolysis due to disease caused by Severe acute respiratory syndrome coronavirus 2 | 3655977 | Clinical Finding | Condition | SNOMED |
| Acute hypoxemic respiratory failure due to disease caused by Severe acute respiratory syndrome coronavirus 2 | 3655976 | Clinical Finding | Condition | SNOMED |
| Sepsis due to disease caused by Severe acute respiratory syndrome coronavirus 2 | 3655975 | Clinical Finding | Condition | SNOMED |
| Respiratory infection caused by COVID-19 | 756039 | Clinical Finding | Condition | OMOP Extension |
| Bronchitis caused by COVID-19 | 756031 | Clinical Finding | Condition | OMOP Extension |
| Patient meets COVID-19 laboratory diagnostic criteria | 704996 | Clinical Finding | Observation | OMOP Extension |
| Patient meets COVID-19 clinical diagnostic criteria | 704995 | Clinical Finding | Observation | OMOP Extension |
| Patient meets COVID-19 laboratory confirmation criterion (detection of specific RNA in a clinical specimen using a molecular amplification detection test) | 700297 | Clinical Finding | Observation | OMOP Extension |
| Patient meets COVID-19 presumptive laboratory evidence criteria (detection of specific antigen in a clinical specimen, OR detection of specific antibody in serum, plasma, or whole blood indicative of a new or recent infection) | 700296 | Clinical Finding | Observation | OMOP Extension |
| Coronavirus infection | 439676 | Clinical Finding | Condition | SNOMED |
| Severe acute respiratory syndrome | 320651 | Clinical Finding | Condition | SNOMED |

**Table S4.** Concept ids for negative control outcomes

| **Negative control outcome** | **id** | **Class** | **Domain** | **Vocabulary** |
| --- | --- | --- | --- | --- |
| Acid reflux | 44783954 | Clinical Finding | Condition | SNOMED |
| Acquired hypothyroidism | 138384 | Clinical Finding | Condition | SNOMED |
| Actinic keratosis | 138825 | Clinical Finding | Condition | SNOMED |
| Acute conjunctivitis | 376707 | Clinical Finding | Condition | SNOMED |
| Age related macular degeneration | 374028 | Clinical Finding | Condition | SNOMED |
| Basal cell carcinoma of skin | 4112752 | Clinical Finding | Condition | SNOMED |
| Benign prostatic hyperplasia | 198803 | Clinical Finding | Condition | SNOMED |
| Bilateral cataracts | 4317977 | Clinical Finding | Condition | SNOMED |
| Blepharitis | 378425 | Clinical Finding | Condition | SNOMED |
| Cataract | 375545 | Clinical Finding | Condition | SNOMED |
| Cellulitis of lower limb | 42709838 | Clinical Finding | Condition | SNOMED |
| Constipation | 75860 | Clinical Finding | Condition | SNOMED |
| Dry eyes | 4036620 | Clinical Finding | Condition | SNOMED |
| Foot pain | 4169905 | Clinical Finding | Condition | SNOMED |
| Gallstone | 196456 | Clinical Finding | Condition | SNOMED |
| Glaucoma | 437541 | Clinical Finding | Condition | SNOMED |
| Hearing difficulty | 4038030 | Clinical Finding | Condition | SNOMED |
| Hearing loss | 377889 | Clinical Finding | Condition | SNOMED |
| Hemorrhoids | 195562 | Clinical Finding | Condition | SNOMED |
| Hypothyroidism | 140673 | Clinical Finding | Condition | SNOMED |
| Impacted cerumen | 374375 | Clinical Finding | Condition | SNOMED |
| Inguinal hernia | 4288544 | Clinical Finding | Condition | SNOMED |
| Intraocular pressure left eye | 4217260 | Clinical Finding | Condition | SNOMED |
| Iron deficiency anemia | 436659 | Clinical Finding | Condition | SNOMED |
| Laceration - injury | 443419 | Clinical Finding | Condition | SNOMED |
| Laceration of lower leg | 4155040 | Clinical Finding | Condition | SNOMED |
| Open wound of lower leg | 4053604 | Clinical Finding | Condition | SNOMED |
| Osteopenia | 4195039 | Clinical Finding | Condition | SNOMED |
| Otitis externa | 380731 | Clinical Finding | Condition | SNOMED |
| Polyp of colon | 4285898 | Clinical Finding | Condition | SNOMED |
| Pressure ulcer | 135333 | Clinical Finding | Condition | SNOMED |
| Prostatism | 4016155 | Clinical Finding | Condition | SNOMED |
| Rectal hemorrhage | 4026112 | Clinical Finding | Condition | SNOMED |
| Senile hyperkeratosis | 141932 | Clinical Finding | Condition | SNOMED |
| Squamous cell carcinoma of skin | 4111921 | Clinical Finding | Condition | SNOMED |
| Traumatic wound | 46287159 | Clinical Finding | Condition | SNOMED |
| Ulcer of foot | 74719 | Clinical Finding | Condition | SNOMED |
| Ulcer of lower extremity | 197304 | Clinical Finding | Condition | SNOMED |
| Urinary incontinence | 197672 | Clinical Finding | Condition | SNOMED |
| Vaginal irritation | 4058568 | Clinical Finding | Condition | SNOMED |
| Vitamin D deficiency | 436070 | Clinical Finding | Condition | SNOMED |
| Vulval irritation | 4060207 | Clinical Finding | Condition | SNOMED |
| Wax in ear canal | 4155902 | Clinical Finding | Condition | SNOMED |

1. **Target trial emulation**

**Table S5. Design characteristics** of Phase 3 Vaccine efficacy randomized controlled trials (RCT) and observational target trial emulation study

|  | **BNT162b2**  **(Polack et al. NEJM)** | **ChAdOx1**  **(Voysey et al. Lancet)** | **Our study** | **Comment** |
| --- | --- | --- | --- | --- |
| **Comparator** | Placebo | Meningococcal group A, C, W, and Y conjugate vaccine or saline | Non-vaccinated people |  |
| **Study time** | 27^th^ July - Nov 14^th^, 2020 | 23^th^ April - Nov 4^th^, 2020 | January 2021 | At the time of RCT, vaccines did not yet have marketing authorisation. We started our study as soon as the vaccination rollout started in the community. |
| **Study population** | Adults ≥16 years, who were healthy or had stable chronic medical conditions | Participants aged ≥18 years | Participants aged ≥75 |  |
| *Age* | Median: 52 Range: (16-89) | Predominantly 18–55 (79-100%) | Median 78-82 | We prioritised synchronising calendar time for our trial emulation, as previous studies found high variability in vaccine response for variants. This allowed us to as good as possible match the predominant variant, herd immunity in the population and social distancing tiers as close as possible to RCT. |
| *Gender* | 51.1% male | 57-67% male | 42.1%-44.3% male |  |
| *BMI* | 34.8% obese | Median 25.2 – 25.6 | NA |  |
| **Exclusion criteria** | - Previous SARS-CoV-2 infection - Treatment with immunosuppressive therapy - Diagnosis with immune-comprising condition | - Previous SARS-CoV-2 infection (seropositive at baseline) - People with NAAT-positive swabs within 14 days after the second vaccination. - Follow-up time of <15 days after the second vaccination - Individuals with NAAT-positive swab within 21 days after their first standard-dose vaccine | - Previous SARS-CoV-2 infection - Previous vaccination - For ChAdOx1: individuals who tested positive or were censored before the 21th day were eliminated from the analysis | Previous vaccination was no explicit exclusion criterion in the trial, but people could not have been vaccinated before as no vaccines were approved back then. |
| **Outcome definition** | Laboratory-confirmed, symptomatic Covid-19 (fever, new or increased cough, new or increased shortness of breath, chills, new or increased muscle pain, new loss of taste or smell, sore throat, diarrhea, or vomiting with positive SARS-CoV-2 test) | Primary symptomatic COVID-19, with fever of at least 37·8°C, cough, shortness of breath, and anosmia or ageusia (UK definition) | Positive PCR test, AND Positive PCR test /clinical diagnosis of COVID-19 (closer to the trial definition) |  |
| **Analysis for replication: Follow-up** | After dose 1 to before dose 2 (21days): 52.4 (29.5–68.4) | >21 days after the 1 standard dose in seronegative participants who received only standard doses 64·1% (50·5 to 73·9) | BNT162b2: Day 1 – 21 with censoring at 2^nd^ vaccine dose  ChAdOx1: Day 21 – 12weeks, with censoring at 2^nd^ vaccine dose |  |


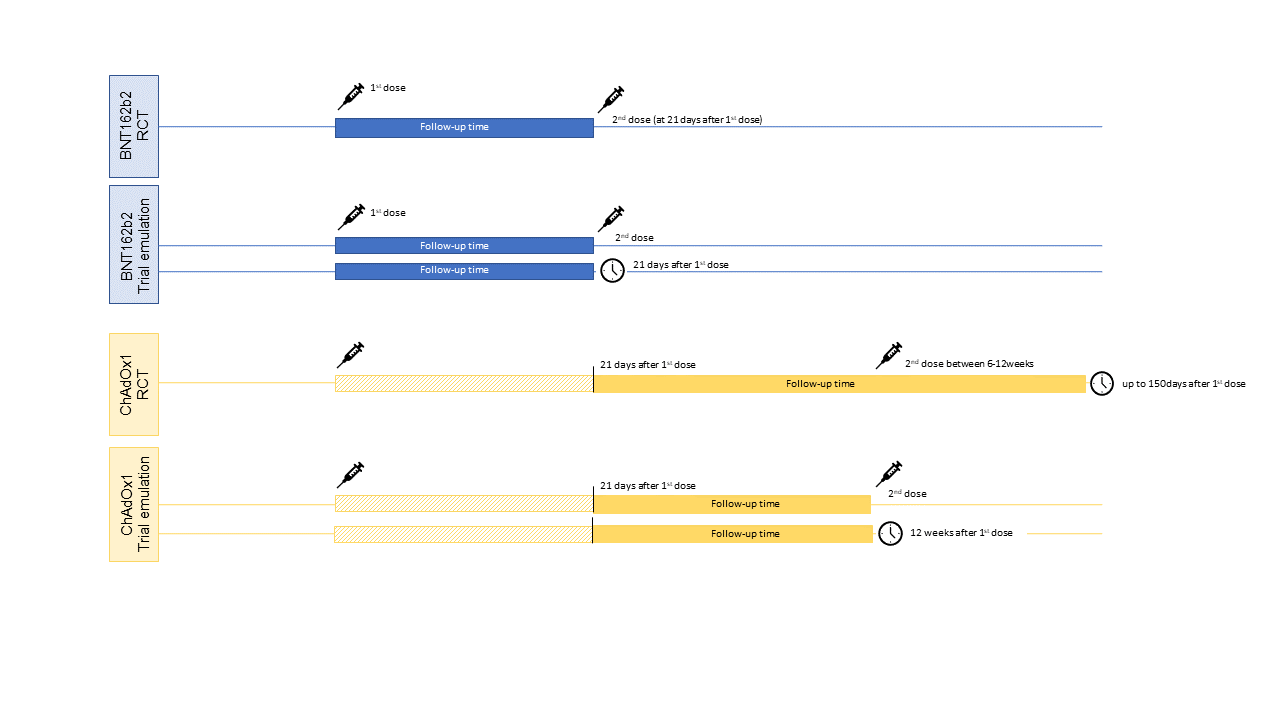


**Figure S1**. Mode of follow-up for RCT vs. observational study

*For ChAdOx1 RCT: At 90days after first vaccine dose, 58% of people who received a first vaccine dose (ChAdOx1 or comparator) were still under observation.

1. **Vaccine specific analyses**
   1.
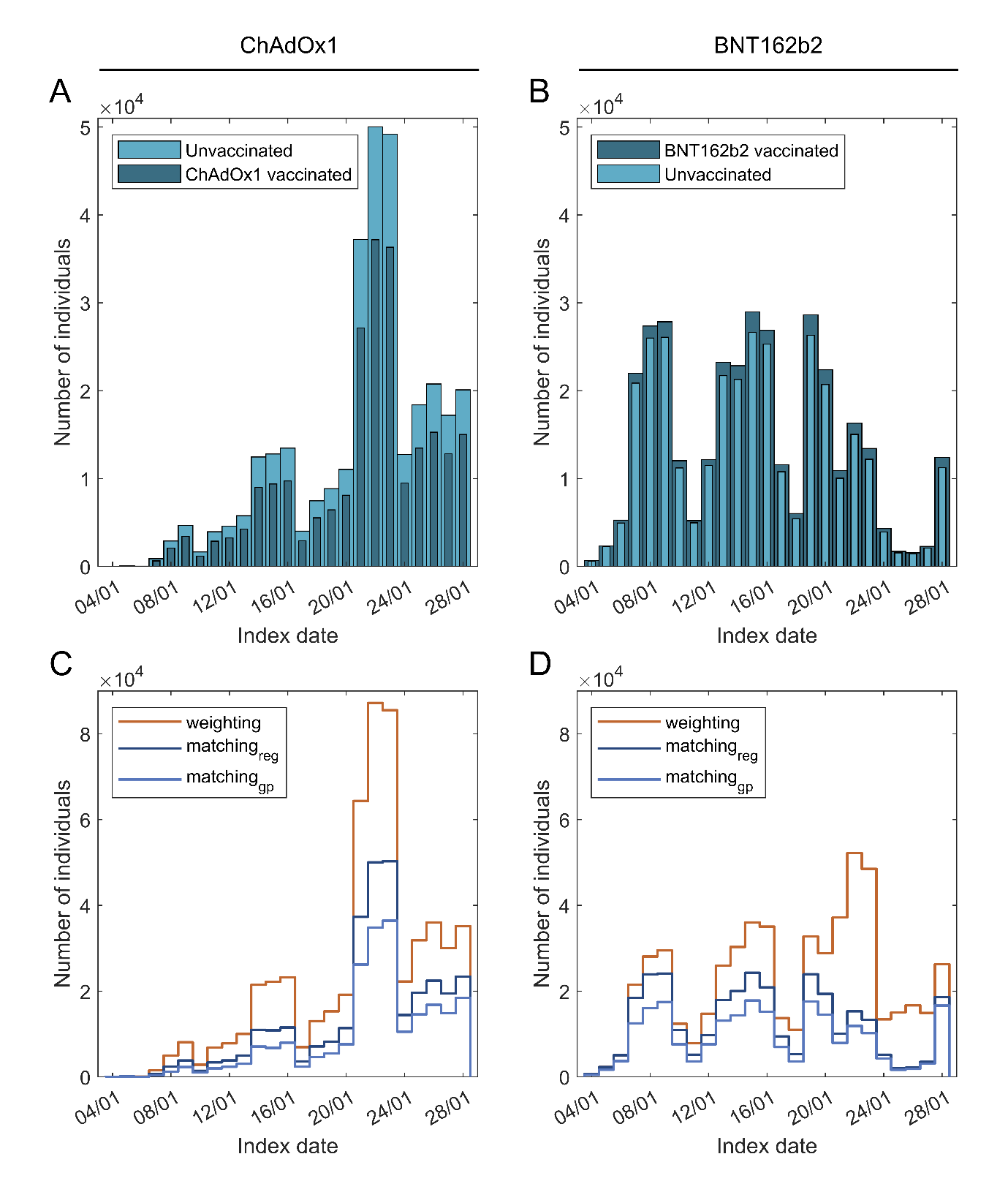
**Index dates**

**Figure S2**. Index date distribution. (A) ChAdOx1 – unvaccinated comparison. (B) BNT162b2 – unvaccinated comparison. (C) ChAdOx1 – unvaccinated weighted and matched cohorts. (D) BNT162b2 – unvaccinated weighted and matched cohorts. matching_gp_: GP practice was used for matching based on location; matching_reg_: region was used for matching based on location.

- 1. **
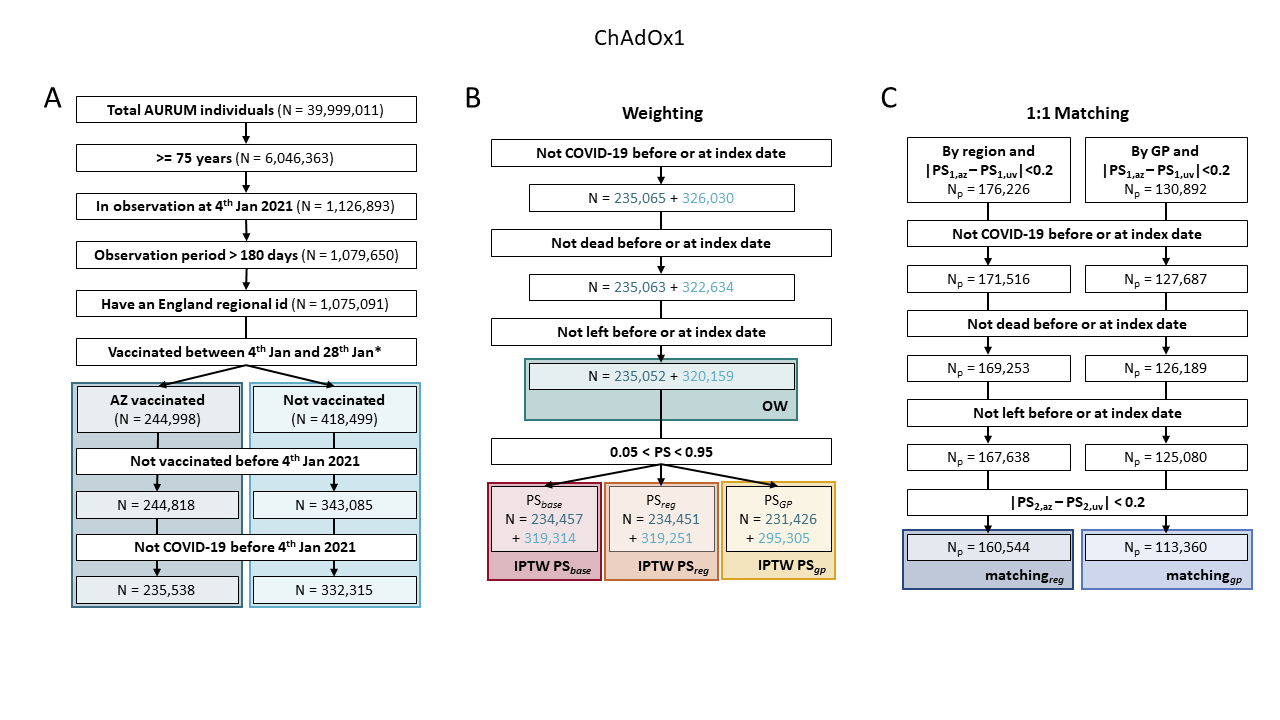
Cohort counts**

**Figure S3. Cohorts building flowchart in ChAdOx1 and unvaccinated comparison. (A)** Flowchart to build the ChAdOx1 vaccinated and unvaccinated initial cohorts. **(B)** Flowchart to build the different weighting cohorts, the start point of these cohorts is the end of panel A. Dark blue numbers are for ChAdOx1 vaccinated cohort and light for unvaccinated. PS_base:_ propensity scores were computed without location, PS_reg:_ PS were computed with region included as covariate; PS_gp:_ PS included GP practice as covariate. **(C)** Flowchart to build the different matching cohorts, the start point of these cohorts is the end of panel A. PS_1_ and PS_2_ are the propensity scores (PS) computed at the start and index date, respectively. *At this step individuals with a record of both ChAdOx1 and BNT162b2 vaccines at the index date were excluded.


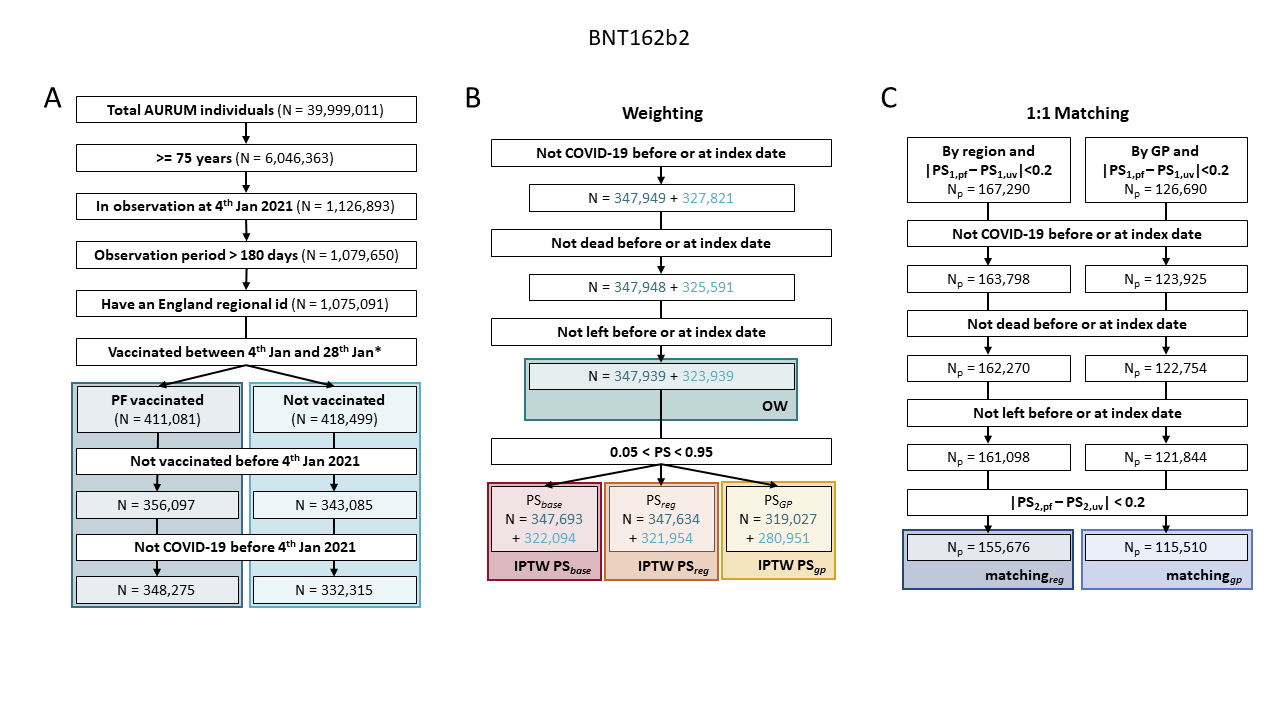


**Figure S4. Cohorts building flowchart in BNT162b2 and unvaccinated comparison. (A)** Flowchart to build the BNT162b2 vaccinated and unvaccinated initial cohorts. **(B)** Flowchart to build the different weighting cohorts, the start point of these cohorts is the end of panel A. Dark blue numbers are for BNT162b2 vaccinated cohort and light for unvaccinated. PS_base:_ propensity scores were computed without location, PS_reg:_ PS were computed with region included as covariate; PS_gp:_ PS included GP practice as covariate. **(C)** Flowchart to build the different matching cohorts, the start point of these cohorts is the end of panel A. PS_1_ and PS_2_ are the propensity scores (PS) computed at the start and index date, respectively. *At this step individuals with a record of both ChAdOx1 and BNT162b2 vaccines at the index date were excluded.

- 1. **Standardized mean differences**

**Table S6. Standardized mean differences (SMDs) unvaccinated vs vaccinated comparison before PS weighting/matching.** Only the 23 covariates with standardized mean differences greater than 0.1 are shown. 3 different periods are defined: short (-30 to -1 respect index date), mid (-180 to -31 from index date) and long (from any time prior to -181 days from index date).

| **Covariate** | **SMD** | **Vaccinated**  **mean ± STD** | **Unvaccinated**  **mean ± STD** |
| --- | --- | --- | --- |
| General Practice centre | 0.74 | categorical | |
| Age group | 0.53 | categorical | |
| Age | 0.31 | 82.2±5.4 | 79.9±5.6 |
| No response to bowel cancer screening invitation (*long*) | 0.28 | 0.19±0.39 | 0.31±0.46 |
| Normal test on bowel cancer screening programme (*long*) | 0.17 | 0.39±0.49 | 0.47±0.50 |
| Pulse rate measurement (*long*) | 0.16 | 0.88±0.32 | 0.82±0.38 |
| Diastolic blood pressure reading (*long*) | 0.15 | 1.00±0.06 | 0.98±0.14 |
| Systolic blood pressure reading (*long*) | 0.15 | 1.00±0.06 | 0.98±0.14 |
| Blood pressure reading (*long*) | 0.15 | 1.00±0.06 | 0.98±0.14 |
| Region | 0.14 | categorical | |
| Number of visits to the GP (*long*) | 0.14 | 359±216 | 317±217 |
| Normal test on bowel cancer screening programme (*mid*) | 0.12 | 0.02±0.14 | 0.04±0.19 |
| Diastolic blood pressure reading (m*id*) | 0.12 | 0.36±0.48 | 0.30±0.46 |
| Systolic blood pressure reading (*mid*) | 0.12 | 0.36±0.48 | 0.30±0.46 |
| Blood pressure reading (*mid*) | 0.12 | 0.35±0.48 | 0.30±0.46 |
| Skin lesion (*long*) | 0.12 | 0.29±0.46 | 0.24±0.43 |
| Actinic keratosis (*long*) | 0.12 | 0.15±0.35 | 0.11±0.31 |
| Number of visits to the GP (*short*) | 0.11 | 2.28±2.38 | 1.89±2.41 |
| Wax in ear canal (*long*) | 0.11 | 0.26±0.44 | 0.21±0.41 |
| Senile hyperkeratosis (*long*) | 0.10 | 0.23±0.42 | 0.19±0.39 |
| Chronic kidney disease stage 3 (*long*) | 0.10 | 0.21±0.41 | 0.17±0.37 |
| Moderate frailty (*long*) | 0.10 | 0.16±0.37 | 0.13±0.33 |
| Basal cell carcinoma of skin (*long*) | 0.10 | 0.11±0.31 | 0.08±0.27 |

STD: standard deviation

**Table S7. Standardized mean differences (SMDs) ChAdOx1 vaccinated vs unvaccinated comparison before PS weighting/matching.** Only the 25 covariates with standardized mean differences greater than 0.1 are shown. 3 different periods are defined: short (-30 to -1 respect index date), mid (-180 to -31 from index date) and long (from any time prior to -181 days from index date).

| **Covariate** | **SMD** | **ChAdOx1 Vaccinated**  **mean ± STD** | **Unvaccinated**  **mean ± STD** |
| --- | --- | --- | --- |
| General Practice centre | 0.82 | categorical | |
| Age group | 0.33 | categorical | |
| Age | 0.23 | 81.7±5.7 | 79.8±5.6 |
| No response to bowel cancer screening invitation (*long*) | 0.21 | 0.22±0.42 | 0.31±0.46 |
| Region | 0.20 | categorical | |
| Number of any covid-19 test (*mid*) | 0.18 | 0.31±1.13 | 0.08±0.55 |
| Number of PCR test (*mid*) | 0.17 | 0.23±0.89 | 0.06±0.44 |
| Pulse rate measurement (*long*) | 0.15 | 0.88±0.33 | 0.82±0.38 |
| Number of any covid-19 test (*short*) | 0.15 | 0.10±0.44 | 0.03±0.22 |
| Number of PCR test (*short*) | 0.15 | 0.10±0.43 | 0.03±0.21 |
| Diastolic blood pressure reading (*long*) | 0.14 | 1.00±0.07 | 0.98±0.14 |
| Systolic blood pressure reading (*long*) | 0.14 | 1.00±0.07 | 0.98±0.14 |
| Blood pressure reading (*long*) | 0.13 | 1.00±0.07 | 0.98±0.14 |
| Number of visits to the GP (*long*) | 0.13 | 356±219 | 317±217 |
| Number of visits to the GP (*mid*) | 0.11 | 11.8±9.2 | 10.4±9.0 |
| Sever frailty (*long*) | 0.11 | 0.09±0.28 | 0.06±0.24 |
| Skin lesion (*long*) | 0.11 | 0.29±0.45 | 0.24±0.43 |
| Number of visits to the GP (*short*) | 0.11 | 2.29±2.43 | 1.92±2.41 |
| Normal test on bowel cancer screening programme (*long*) | 0.11 | 0.02±0.15 | 0.04±0.20 |
| Blood pressure reading (*mid*) | 0.11 | 0.34±0.48 | 0.30±0.46 |
| Diastolic blood pressure reading (*mid*) | 0.10 | 0.35±0.48 | 0.30±0.46 |
| Systolic blood pressure reading (*mid*) | 0.10 | 0.35±0.48 | 0.30±0.46 |
| Moderate frailty (*long*) | 0.10 | 0.16±0.37 | 0.13±0.33 |
| Alzheimer's disease (*long*) | 0.10 | 0.04±0.20 | 0.02±0.15 |
| Bowels: fully continent (*long*) | 0.10 | 0.07±0.26 | 0.05±0.21 |

STD: standard deviation

**Table S8. Standardized mean differences (SMDs) BNT162b2 vaccinated vs unvaccinated comparison before PS weighting/matching.** Only the 27 covariates with standardized mean differences greater than 0.1 are shown. 3 different periods are defined: short (-30 to -1 respect index date), mid (-180 to -31 from index date) and long (from any time prior to -181 days from index date). STD: standard deviation

| **Covariate** | **SMD** | **BNT162b2 Vaccinated**  **mean ± STD** | **Unvaccinated**  **mean ± STD** |
| --- | --- | --- | --- |
| General Practice centre | 0.99 | categorical | |
| Age group | 0.67 | categorical | |
| Age | 0.36 | 82.6+-5.2 | 79.9+-5.6 |
| No response to bowel cancer screening invitation (*long*) | 0.33 | 0.17+-0.38 | 0.31+-0.46 |
| Normal test on bowel cancer screening programme (*long*) | 0.23 | 0.36+-0.48 | 0.47+-0.50 |
| Pulse rate measurement (*long*) | 0.17 | 0.88+-0.32 | 0.82+-0.38 |
| Blood pressure reading (*long*) | 0.15 | 1.00+-0.06 | 0.98+-0.14 |
| Diastolic blood pressure reading (*long*) | 0.15 | 1.00+-0.06 | 0.98+-0.14 |
| Systolic blood pressure reading (*long*) | 0.15 | 1.00+-0.06 | 0.98+-0.14 |
| Region | 0.15 | categorical | |
| Number of visits to the GP (*long*) | 0.15 | 362+-214 | 318+-217 |
| Actinic keratosis (*long*) | 0.14 | 0.15+-0.36 | 0.11+-0.31 |
| Normal test on bowel cancer screening programme (*mid*) | 0.13 | 0.02+-0.13 | 0.04+-0.19 |
| Diastolic blood pressure reading (m*id*) | 0.13 | 0.36+-0.48 | 0.30+-0.46 |
| Systolic blood pressure reading (*mid*) | 0.13 | 0.36+-0.48 | 0.30+-0.46 |
| Blood pressure reading (*mid*) | 0.13 | 0.36+-0.48 | 0.30+-0.46 |
| Skin lesion (*long*) | 0.13 | 0.30+-0.46 | 0.24+-0.43 |
| Wax in ear canal (*long*) | 0.13 | 0.26+-0.44 | 0.21+-0.41 |
| Number of visits to the GP (*short*) | 0.12 | 2.27+-2.34 | 1.85+-2.41 |
| Senile hyperkeratosis (*long*) | 0.12 | 0.24+-0.42 | 0.19+-0.39 |
| Pulse rhythm regular (*long*) | 0.12 | 0.67+-0.47 | 0.61+-0.49 |
| Basal cell carcinoma of skin (*long*) | 0.11 | 0.11+-0.32 | 0.08+-0.27 |
| Chronic kidney disease stage 3 (*long*) | 0.11 | 0.21+-0.41 | 0.17+-0.37 |
| Cataract (*long*) | 0.11 | 0.16+-0.36 | 0.12+-0.32 |
| Hearing loss (*long*) | 0.11 | 0.19+-0.39 | 0.15+-0.35 |
| Diverticular disease (*long*) | 0.10 | 0.14+-0.35 | 0.11+-0.31 |
| Moderate frailty (*long*) | 0.10 | 0.16+-0.37 | 0.13+-0.33 |


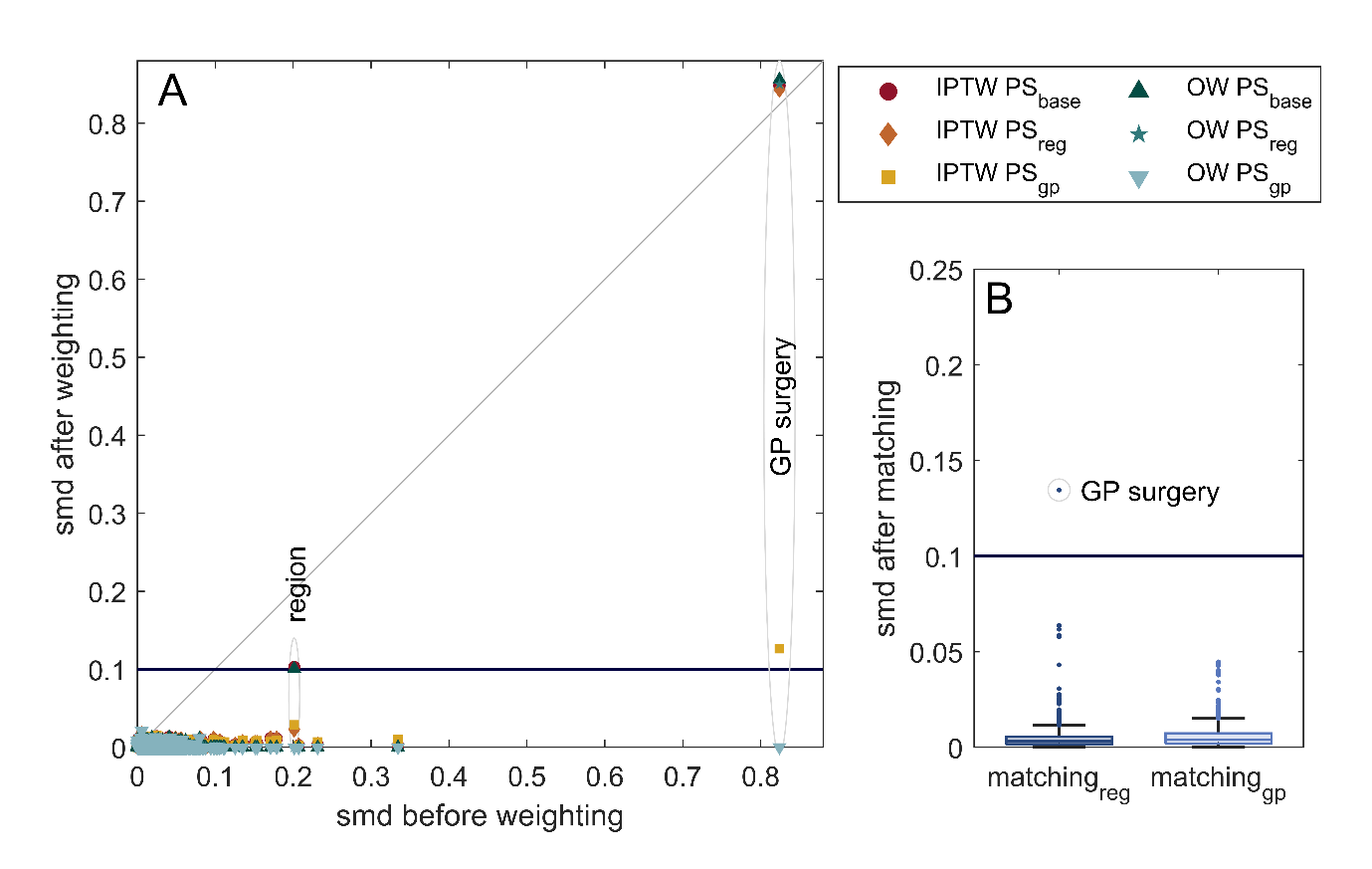
**Figure S5. Standardized mean differences (SMD) for the different methods in ChAdOx1 vaccinated and unvaccinated comparison.** (A) Scatter plot to compare covariate smds before and after PS weighting. Region and GP surgery are the only covariates that often remained unbalanced after weighting. (B) Boxplot for covariate smd after matching. Only GP surgery remained unbalanced after regional matching. SMD: standardized mean difference, IPTW: Inverse probability treatment weighting, OW: Overlap weighting, PS: Propensity Score, GP: General practitioner, Representations of location included in PS: “base”: without location, “region”: Region, “GP”: de-identified GP surgery.


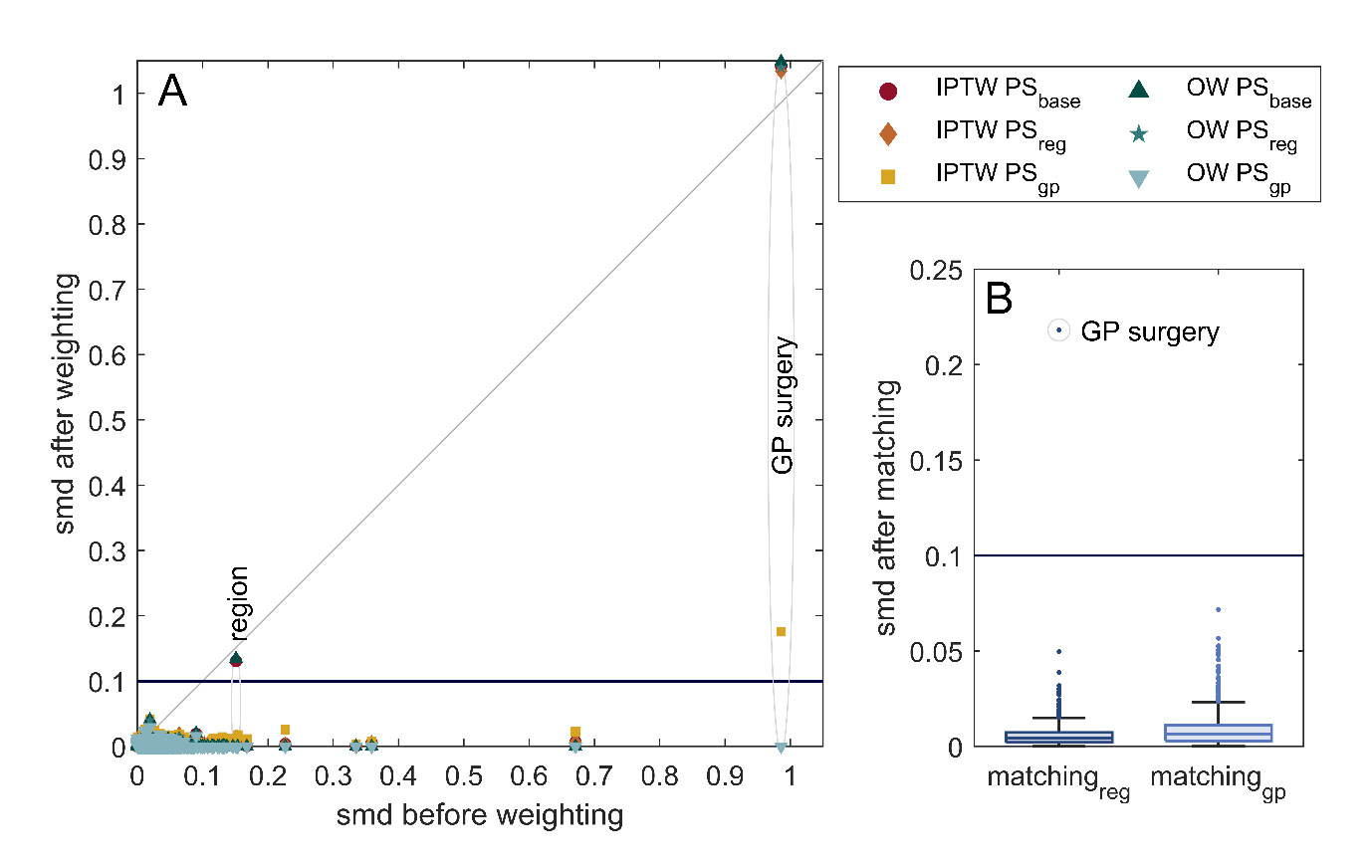


**Figure S6. Standardized mean differences (SMD) for the different methods in BNT162b2 vaccinated and unvaccinated comparison.** (A) Scatter plot to compare covariate smds before and after PS weighting. Region and GP surgery are the only covariates that often remained unbalanced after weighting. (B) Boxplot for covariate smd after matching. Only GP surgery remained unbalanced after regional matching. SMD: standardized mean difference, IPTW: Inverse probability treatment weighting, OW: Overlap weighting, PS: Propensity Score, GP: General practitioner, Representations of location included in PS: “base”: without location, “region”: Region, “GP”: de-identified GP surgery.

- 1. **
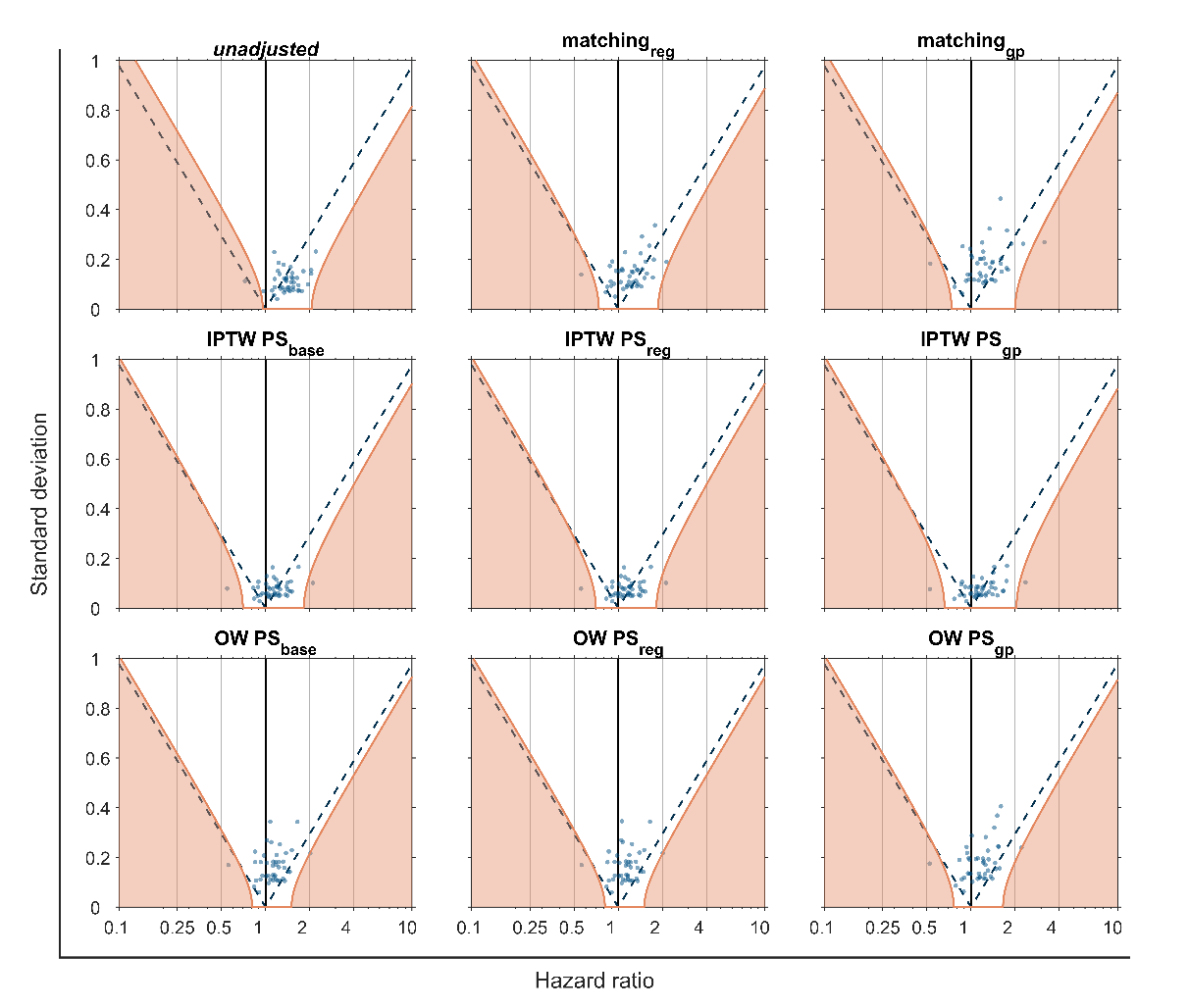
Negative Control Outcomes distribution**

**Figure S7. Negative control outcomes (NCO) hazard ratios and standard deviation for the ChAdOx1 – unvaccinated comparison.** Each blue dot represents a different NCO. Purple dashed lines indicate the significative threshold for the NCO; they are positively correlated if they are on the right of the dashed line and negatively on the left. Orange lines mark significance thresholds after calibration, where we adjust the significant thresholds according to the negative control outcome distribution.

IPTW: Inverse probability treatment weighting, OW: Overlap weighting, PS: Propensity Score. Representations of location included in PS: “base”: without location, “region”: Region, “GP”: de-identified GP surgery.


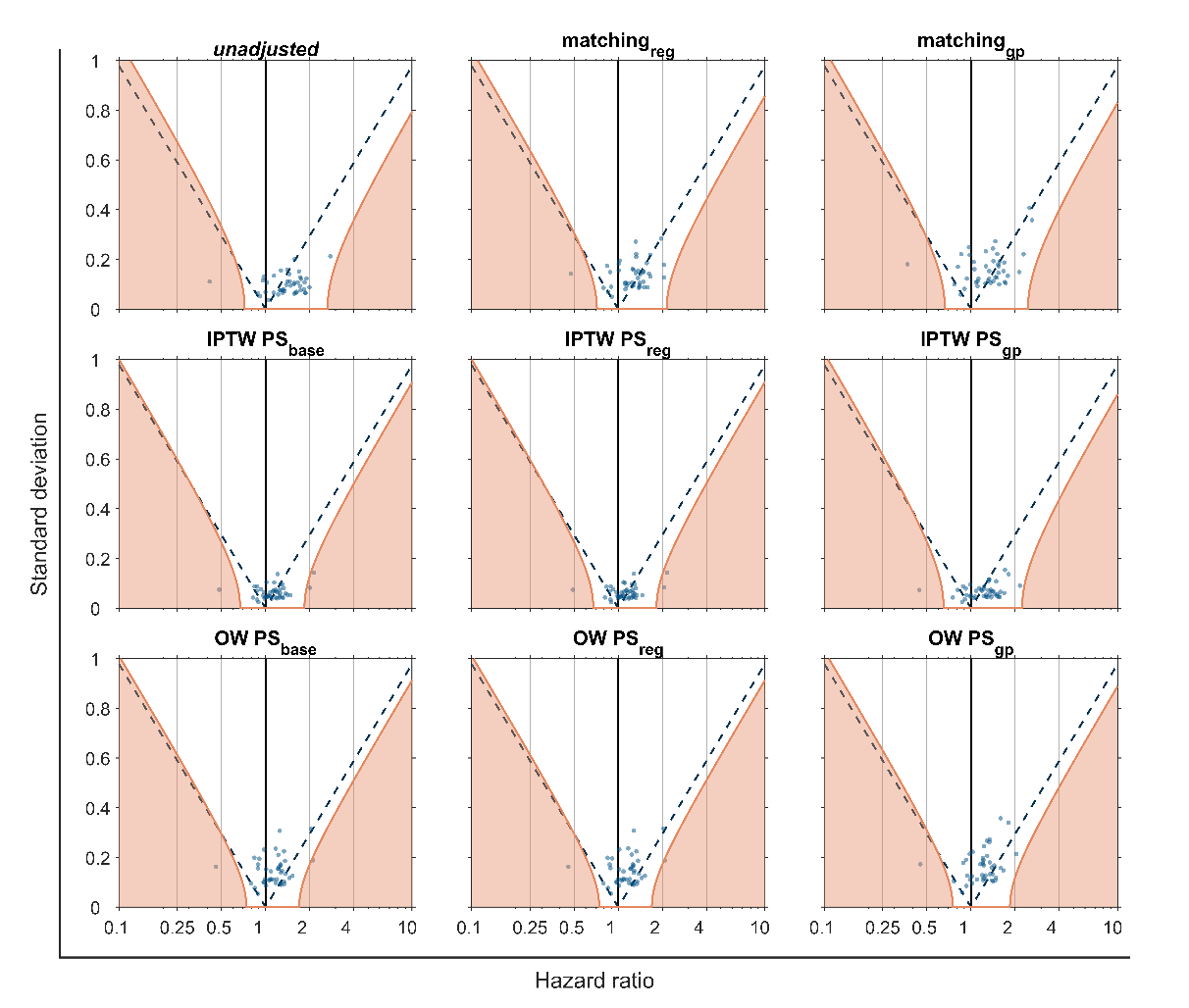


**Figure S8. Negative control outcomes (NCO) hazard ratios and standard deviation for the BNT162b2 – unvaccinated comparison.** Each blue dot represents a different NCO. Purple dashed lines indicate the significative threshold for the NCO; they are positively correlated if they are on the right of the dashed line and negatively on the left. Orange lines mark significance thresholds after calibration, where we adjust the significant thresholds according to the negative control outcome distribution.

IPTW: Inverse probability treatment weighting, OW: Overlap weighting, PS: Propensity Score. Representations of location included in PS: “base”: without location, “region”: Region, “GP”: de-identified GP surgery.

1. **Hazard ratio values**

**
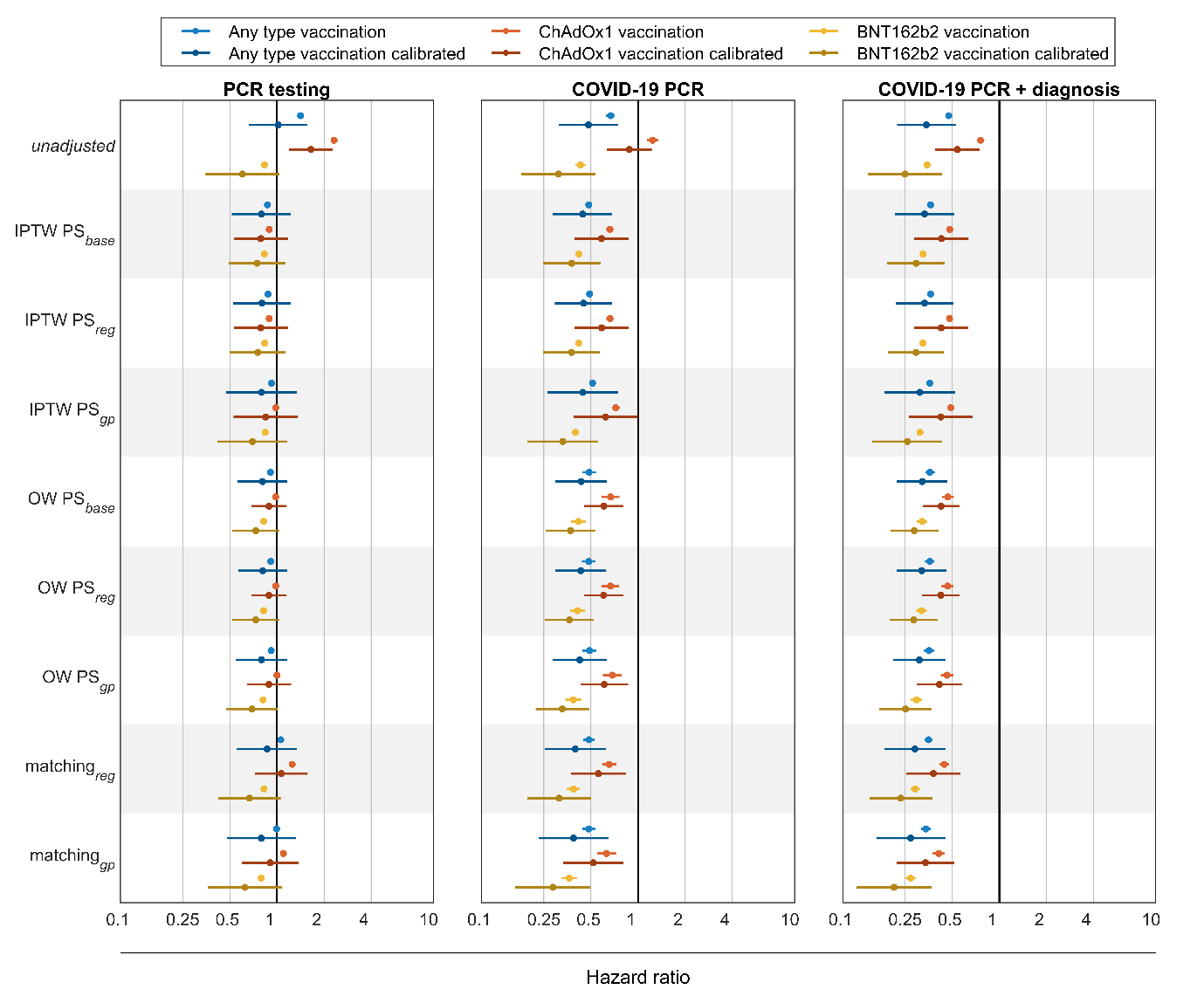
**

**Figure S9. Hazard ratio for the control outcomes during all the follow-up**. Each dot is the hazard ratio (HR) for a different adjustment computed with a Cox proportional hazards regression. Blue lines are for any type of vaccination compared to unvaccinated, red ones for ChAdOx1 vaccinated compared to unvaccinated and yellow lines are for BNT162b2 vaccinated compared to unvaccinated. Darker lines are for calibrated hazard ratios. Vertical black line marks the HR = 1 threshold. In the left panel HR are for PCR testing, central and right panel for different COVID-19 definitions: only PCR positive and PCR positive or a diagnose, respectively.

IPTW: Inverse probability treatment weighting, OW: Overlap weighting, PS: Propensity Score, PCR: Polymerase Chain Reaction, GP: General practitioner, Representations of location included in PS: “base”: without location, “region”: Region, “GP”: de-identified GP surgery, ChAdOx1 and BNT162b2: COVID-19 vaccines.

**Table S9. Hazard ratio (HR) values corresponding to figure 5.** Hazard ratios were computed with a Cox proportional hazards regression censoring at day 10.

| **method** | **calibration** | **Hazard ratios vaccinated** | | |
| --- | --- | --- | --- | --- |
|  |  | **Any** | **ChAdOx1** | **BNT162b2** |
|  |  | **HR [95%CI]** | **HR [95%CI]** | **HR [95%CI]** |
| **PCR testing** | | | | |
| *unadjusted* | No | 1.50 [1.44-1.55] | 2.82 [2.70-2.94] | 0.78 [0.74-0.82] |
|  | Yes | 1.08 [0.70-1.66] | 2.00 [1.45-2.77] | 0.56 [0.33-0.97] |
| IPTW PS_base_ | No | 0.84 [0.82-0.86] | 0.94 [0.92-0.97] | 0.73 [0.71-0.76] |
|  | Yes | 0.76 [0.49-1.18] | 0.83 [0.56-1.24] | 0.66 [0.43-1.01] |
| IPTW PS_reg_ | No | 0.84 [0.82-0.86] | 0.94 [0.92-0.97] | 0.74 [0.72-0.76] |
|  | Yes | 0.77 [0.51-1.18] | 0.83 [0.56-1.24] | 0.67 [0.44-1.01] |
| IPTW PS_gp_ | No | 0.88 [0.86-0.90] | 1.05 [1.02-1.08] | 0.77 [0.75-0.80] |
|  | Yes | 0.76 [0.45-1.28] | 0.91 [0.57-1.45] | 0.64 [0.38-1.07] |
| OW PS_base_ | No | 0.84 [0.80-0.90] | 1.01 [0.94-1.08] | 0.74 [0.69-0.79] |
|  | Yes | 0.75 [0.52-1.09] | 0.91 [0.70-1.19] | 0.66 [0.46-0.94] |
| OW PS_reg_ | No | 0.85 [0.80-0.90] | 1.01 [0.94-1.08] | 0.73 [0.69-0.79] |
|  | Yes | 0.75 [0.52-1.08] | 0.91 [0.70-1.19] | 0.66 [0.46-0.93] |
| OW PS_gp_ | No | 0.86 [0.81-0.92] | 1.02 [0.95-1.09] | 0.75 [0.70-0.81] |
|  | Yes | 0.75 [0.51-1.10] | 0.91 [0.65-1.26] | 0.64 [0.43-0.93] |
| matching_reg_ | No | 1.05 [0.99-1.10] | 1.36 [1.28-1.44] | 0.76 [0.71-0.80] |
|  | Yes | 0.85 [0.55-1.33] | 1.16 [0.78-1.71] | 0.61 [0.39-0.97] |
| matching_gp_ | No | 1.00 [0.94-1.06] | 1.18 [1.09-1.28] | 0.73 [0.68-0.78] |
|  | Yes | 0.80 [0.48-1.32] | 0.97 [0.64-1.49] | 0.57 [0.33-0.99] |
| **COVID-19 PCR** | | | | |
| *unadjusted* | No | 0.52 [0.48-0.57] | 1.03 [0.92-1.16] | 0.31 [0.28-0.34] |
|  | Yes | 0.38 [0.24-0.58] | 0.73 [0.52-1.03] | 0.22 [0.13-0.39] |
| IPTW PS_base_ | No | 0.34 [0.32-0.37] | 0.53 [0.48-0.57] | 0.27 [0.25-0.29] |
|  | Yes | 0.31 [0.20-0.49] | 0.46 [0.31-0.70] | 0.25 [0.16-0.38] |
| IPTW PS_reg_ | No | 0.35 [0.33-0.37] | 0.53 [0.49-0.57] | 0.27 [0.25-0.30] |
|  | Yes | 0.32 [0.21-0.49] | 0.47 [0.31-0.70] | 0.25 [0.16-0.38] |
| IPTW PS_gp_ | No | 0.36 [0.34-0.38] | 0.58 [0.53-0.63] | 0.24 [0.22-0.26] |
|  | Yes | 0.31 [0.19-0.53] | 0.50 [0.31-0.80] | 0.20 [0.12-0.34] |
| OW PS_base_ | No | 0.35 [0.30-0.41] | 0.53 [0.43-0.64] | 0.28 [0.24-0.33] |
|  | Yes | 0.31 [0.21-0.46] | 0.48 [0.35-0.66] | 0.25 [0.17-0.36] |
| OW PS_reg_ | No | 0.35 [0.30-0.41] | 0.53 [0.44-0.64] | 0.27 [0.23-0.33] |
|  | Yes | 0.31 [0.21-0.46] | 0.48 [0.35-0.66] | 0.24 [0.17-0.36] |
| OW PS_gp_ | No | 0.35 [0.30-0.41] | 0.55 [0.45-0.67] | 0.25 [0.21-0.30] |
|  | Yes | 0.31 [0.20-0.46] | 0.49 [0.34-0.72] | 0.21 [0.14-0.32] |
| matching_reg_ | No | 0.36 [0.32-0.41] | 0.46 [0.40-0.54] | 0.26 [0.23-0.31] |
|  | Yes | 0.29 [0.18-0.46] | 0.40 [0.26-0.60] | 0.21 [0.13-0.34] |
| matching_gp_ | No | 0.37 [0.32-0.43] | 0.47 [0.39-0.58] | 0.24 [0.20-0.28] |
|  | Yes | 0.30 [0.18-0.50] | 0.39 [0.25-0.62] | 0.19 [0.11-0.33] |
| **COVID-19 PCR + diagnosis** | | | | |
| *unadjusted* | No | 0.40 [0.38-0.43] | 0.70 [0.64-0.76] | 0.27 [0.25-0.29] |
|  | Yes | 0.29 [0.19-0.45] | 0.50 [0.36-0.69] | 0.20 [0.11-0.34] |
| IPTW PS_base_ | No | 0.28 [0.27-0.30] | 0.42 [0.40-0.45] | 0.24 [0.22-0.25] |
|  | Yes | 0.26 [0.17-0.40] | 0.37 [0.25-0.56] | 0.21 [0.14-0.33] |
| IPTW PS_reg_ | No | 0.28 [0.27-0.30] | 0.42 [0.40-0.45] | 0.24 [0.22-0.25] |
|  | Yes | 0.26 [0.17-0.39] | 0.37 [0.25-0.56] | 0.21 [0.14-0.33] |
| IPTW PS_gp_ | No | 0.28 [0.27-0.30] | 0.44 [0.41-0.46] | 0.22 [0.21-0.24] |
|  | Yes | 0.25 [0.15-0.42] | 0.38 [0.23-0.60] | 0.19 [0.11-0.31] |
| OW PS_base_ | No | 0.29 [0.26-0.32] | 0.40 [0.35-0.46] | 0.24 [0.21-0.27] |
|  | Yes | 0.26 [0.17-0.37] | 0.37 [0.27-0.49] | 0.21 [0.15-0.31] |
| OW PS_reg_ | No | 0.29 [0.25-0.32] | 0.41 [0.35-0.47] | 0.24 [0.21-0.27] |
|  | Yes | 0.25 [0.17-0.37] | 0.37 [0.27-0.49] | 0.21 [0.15-0.30] |
| OW PS_gp_ | No | 0.29 [0.25-0.32] | 0.41 [0.35-0.47] | 0.22 [0.19-0.25] |
|  | Yes | 0.25 [0.17-0.37] | 0.36 [0.26-0.52] | 0.19 [0.13-0.28] |
| matching_reg_ | No | 0.29 [0.26-0.32] | 0.37 [0.33-0.41] | 0.23 [0.21-0.26] |
|  | Yes | 0.24 [0.15-0.37] | 0.31 [0.21-0.47] | 0.19 [0.12-0.30] |
| matching_gp_ | No | 0.29 [0.26-0.32] | 0.37 [0.32-0.43] | 0.20 [0.18-0.23] |
|  | Yes | 0.23 [0.14-0.39] | 0.31 [0.20-0.48] | 0.16 [0.09-0.28] |

HR: Hazard Ratio, 95%CI: 95% confidence interval, IPTW: Inverse probability treatment weighting, OW: Overlap weighting, PS: Propensity Score, PCR: Polymerase Chain Reaction, GP: General practitioner, Representations of location included in PS: “base”: without location, “region”: Region, “GP”: de-identified GP surgery, ChAdOx1 and BNT162b2: COVID-19 vaccines.

**Table S10. Hazard ratio (HR) values corresponding to figure S9.** Hazard ratios were computed with a Cox proportional hazards regression.

| **method** | **calibration** | **Hazard ratios vaccinated** | | |
| --- | --- | --- | --- | --- |
|  |  | **Any** | **ChAdOx1** | **BNT162b2** |
|  |  | **HR [95%CI]** | **HR [95%CI]** | **HR [95%CI]** |
| **PCR testing** | | | | |
| *unadjusted* | No | 1.41 [1.38-1.45] | 2.32 [2.25-2.39] | 0.83 [0.81-0.86] |
|  | Yes | 1.02 [0.66-1.57] | 1.65 [1.19-2.27] | 0.60 [0.35-1.04] |
| IPTW PS_base_ | No | 0.87 [0.86-0.88] | 0.89 [0.88-0.91] | 0.83 [0.82-0.85] |
|  | Yes | 0.80 [0.52-1.23] | 0.79 [0.53-1.18] | 0.75 [0.49-1.14] |
| IPTW PS_reg_ | No | 0.88 [0.86-0.89] | 0.89 [0.88-0.91] | 0.84 [0.82-0.85] |
|  | Yes | 0.80 [0.53-1.22] | 0.79 [0.53-1.17] | 0.75 [0.50-1.14] |
| IPTW PS_gp_ | No | 0.92 [0.91-0.94] | 0.98 [0.96-1.00] | 0.84 [0.83-0.86] |
|  | Yes | 0.80 [0.47-1.34] | 0.85 [0.53-1.35] | 0.70 [0.42-1.17] |
| OW PS_base_ | No | 0.91 [0.88-0.94] | 0.98 [0.94-1.03] | 0.82 [0.79-0.86] |
|  | Yes | 0.81 [0.56-1.17] | 0.89 [0.69-1.15] | 0.73 [0.52-1.04] |
| OW PS_reg_ | No | 0.91 [0.88-0.95] | 0.98 [0.94-1.03] | 0.82 [0.79-0.86] |
|  | Yes | 0.81 [0.56-1.17] | 0.89 [0.69-1.16] | 0.73 [0.52-1.04] |
| OW PS_gp_ | No | 0.92 [0.88-0.95] | 1.00 [0.96-1.04] | 0.82 [0.78-0.85] |
|  | Yes | 0.80 [0.54-1.17] | 0.89 [0.64-1.23] | 0.69 [0.48-1.01] |
| matching_reg_ | No | 1.06 [1.02-1.09] | 1.25 [1.21-1.30] | 0.83 [0.80-0.86] |
|  | Yes | 0.86 [0.55-1.35] | 1.07 [0.72-1.57] | 0.67 [0.42-1.06] |
| matching_gp_ | No | 0.99 [0.96-1.03] | 1.10 [1.05-1.16] | 0.79 [0.76-0.83] |
|  | Yes | 0.80 [0.48-1.32] | 0.91 [0.60-1.38] | 0.62 [0.36-1.08] |
| **COVID-19 PCR** | | | | |
| *unadjusted* | No | 0.67 [0.63-0.71] | 1.24 [1.14-1.34] | 0.43 [0.40-0.46] |
|  | Yes | 0.48 [0.31-0.74] | 0.88 [0.63-1.22] | 0.31 [0.18-0.54] |
| IPTW PS_base_ | No | 0.48 [0.46-0.50] | 0.66 [0.62-0.70] | 0.42 [0.40-0.44] |
|  | Yes | 0.44 [0.29-0.68] | 0.58 [0.39-0.87] | 0.38 [0.25-0.58] |
| IPTW PS_reg_ | No | 0.49 [0.47-0.51] | 0.66 [0.62-0.70] | 0.42 [0.40-0.44] |
|  | Yes | 0.45 [0.29-0.68] | 0.58 [0.39-0.87] | 0.38 [0.25-0.57] |
| IPTW PS_gp_ | No | 0.51 [0.49-0.53] | 0.72 [0.68-0.76] | 0.40 [0.38-0.42] |
|  | Yes | 0.44 [0.26-0.75] | 0.62 [0.39-1.00] | 0.33 [0.20-0.55] |
| OW PS_base_ | No | 0.49 [0.44-0.54] | 0.67 [0.59-0.76] | 0.41 [0.37-0.46] |
|  | Yes | 0.43 [0.30-0.63] | 0.60 [0.45-0.81] | 0.37 [0.26-0.53] |
| OW PS_reg_ | No | 0.48 [0.44-0.54] | 0.67 [0.58-0.76] | 0.41 [0.37-0.46] |
|  | Yes | 0.43 [0.30-0.63] | 0.60 [0.45-0.80] | 0.36 [0.25-0.52] |
| OW PS_gp_ | No | 0.49 [0.44-0.54] | 0.68 [0.60-0.78] | 0.39 [0.34-0.43] |
|  | Yes | 0.42 [0.29-0.63] | 0.61 [0.43-0.86] | 0.33 [0.22-0.49] |
| matching_reg_ | No | 0.48 [0.45-0.53] | 0.65 [0.59-0.73] | 0.39 [0.35-0.43] |
|  | Yes | 0.40 [0.25-0.62] | 0.56 [0.37-0.83] | 0.31 [0.20-0.50] |
| matching_gp_ | No | 0.48 [0.44-0.53] | 0.63 [0.55-0.72] | 0.36 [0.32-0.41] |
|  | Yes | 0.39 [0.23-0.65] | 0.52 [0.33-0.80] | 0.29 [0.16-0.50] |
| **COVID-19 PCR + diagnosis** | | | | |
| *unadjusted* | No | 0.48 [0.45-0.50] | 0.76 [0.72-0.80] | 0.35 [0.33-0.36] |
|  | Yes | 0.34 [0.22-0.53] | 0.54 [0.39-0.75] | 0.25 [0.15-0.43] |
| IPTW PS_base_ | No | 0.36 [0.35-0.38] | 0.48 [0.46-0.50] | 0.33 [0.32-0.34] |
|  | Yes | 0.33 [0.22-0.51] | 0.43 [0.29-0.64] | 0.29 [0.19-0.45] |
| IPTW PS_reg_ | No | 0.36 [0.35-0.38] | 0.48 [0.46-0.50] | 0.33 [0.31-0.34] |
|  | Yes | 0.33 [0.22-0.51] | 0.43 [0.29-0.63] | 0.29 [0.19-0.44] |
| IPTW PS_gp_ | No | 0.36 [0.35-0.37] | 0.49 [0.47-0.51] | 0.31 [0.30-0.32] |
|  | Yes | 0.31 [0.18-0.52] | 0.42 [0.26-0.68] | 0.26 [0.15-0.43] |
| OW PS_base_ | No | 0.36 [0.34-0.39] | 0.47 [0.43-0.51] | 0.32 [0.30-0.35] |
|  | Yes | 0.32 [0.22-0.47] | 0.42 [0.32-0.56] | 0.29 [0.20-0.41] |
| OW PS_reg_ | No | 0.36 [0.33-0.39] | 0.47 [0.43-0.51] | 0.32 [0.29-0.34] |
|  | Yes | 0.32 [0.22-0.46] | 0.42 [0.32-0.56] | 0.28 [0.20-0.40] |
| OW PS_gp_ | No | 0.36 [0.33-0.38] | 0.46 [0.42-0.51] | 0.30 [0.27-0.32] |
|  | Yes | 0.31 [0.21-0.45] | 0.41 [0.30-0.58] | 0.25 [0.17-0.37] |
| matching_reg_ | No | 0.35 [0.33-0.38] | 0.45 [0.41-0.48] | 0.29 [0.27-0.31] |
|  | Yes | 0.29 [0.18-0.45] | 0.38 [0.26-0.56] | 0.23 [0.15-0.37] |
| matching_gp_ | No | 0.34 [0.32-0.36] | 0.41 [0.37-0.45] | 0.27 [0.25-0.29] |
|  | Yes | 0.27 [0.16-0.45] | 0.34 [0.22-0.52] | 0.21 [0.12-0.37] |

HR: Hazard Ratio, 95%CI: 95% confidence interval, IPTW: Inverse probability treatment weighting, OW: Overlap weighting, PS: Propensity Score, PCR: Polymerase Chain Reaction, GP: General practitioner, Representations of location included in PS: “base”: without location, “region”: Region, “GP”: de-identified GP surgery, ChAdOx1 and BNT162b2: COVID-19 vaccines.

**Table S11. Hazard ratio (HR) values and 95% confidence intervals for trial emulation estimates.** Hazard ratios were computed with a Cox proportional hazards regression.

|  | **Trial emulation** | **Estimates** | | | |
| --- | --- | --- | --- | --- | --- |
|  |  | PCR+ | PCR+  calibrated | PCR+ Diag | PCR+ Diag calibrated |
| **ChAdOx1** [10] | 0.36 [0.26-0.50] | 0.85 [0.67-1.08] | 0.76 [0.51-1.12] | 0.48 [0.42-0.56] | 0.43 [0.30-0.60] |
| **BNT162b2** 3 weeks [9] | 0.48 [0.32-0.71] | 0.34 [0.29-0.39] | 0.29 [0.19-0.43] | 0.27 [0.24-0.30] | 0.23 [0.15-0.34] |
| **BNT162b2** 12 weeks [16] | 0.26 | 0.38 [0.34-0.43] | 0.33 [0.22-0.48] | 0.29 [0.27-0.32] | 0.25 [0.17-0.37] |

HR: Hazard Ratio, 95%CI: 95% confidence interval. PCR: Polymerase Chain Reaction, Diag: COVID-19 Diagnosis, ChAdOx1 and BNT162b2: COVID-19 vaccines.


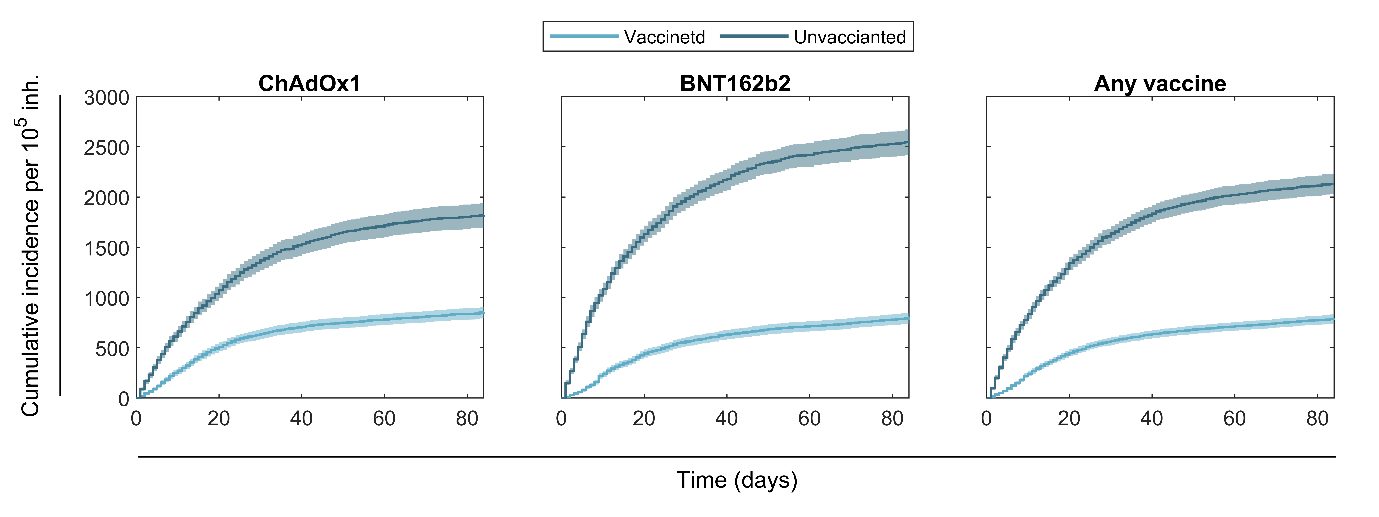
**Figure S10. Kaplan Meier plots for COVID-19**. Kaplan Meier plots using overlap weighting with PS_GP_ for COVID-19 PCR test positive or diagnose definition.


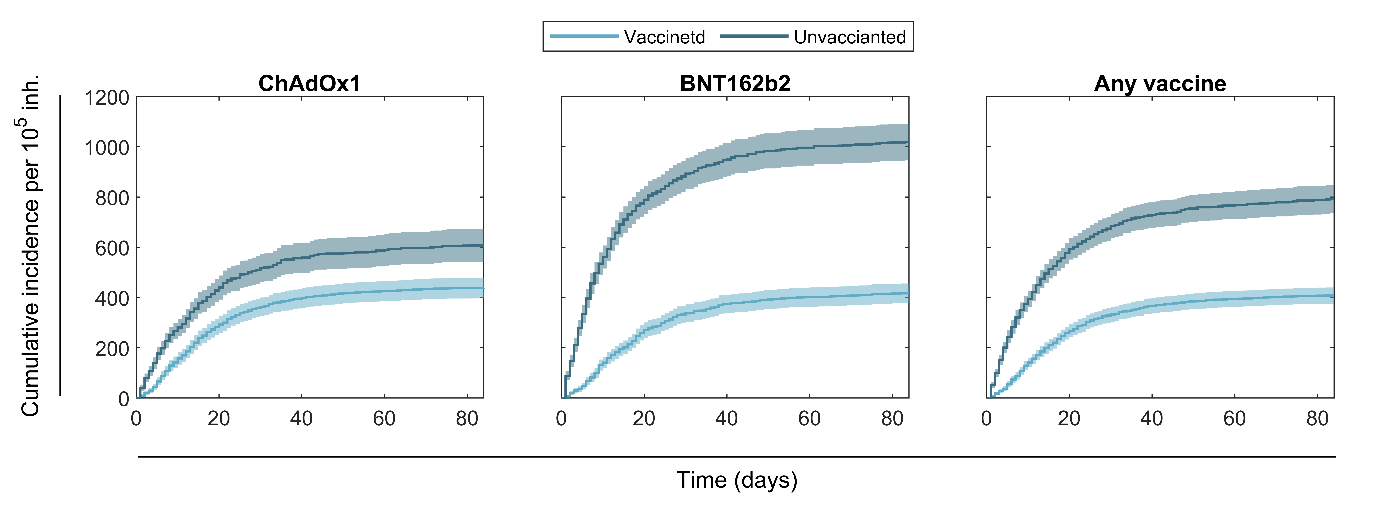


**Figure S11. Kaplan Meier plots for COVID-19**. Kaplan Meier plots using overlap weighting with PS_GP_ for COVID-19 PCR test positive definition.
